# Outcomes of a National, Cross-Sector Antimicrobial Stewardship Training Initiative for Pharmacists in England

**DOI:** 10.3390/pharmacy9040165

**Published:** 2021-10-10

**Authors:** Vincent Ng, Diane Ashiru-Oredope, Helena Rosado, Beth Ward

**Affiliations:** 1 Royal Pharmaceutical Society, 66-68 East Smithfield, London E1W 1AW, UK; vincentnwork@gmail.com (V.N.); helena.rosado@rpharms.com (H.R.); 2 HCAI, Fungal, AMR, AMU & Sepsis Division, UK Health Security Agency, London SE1 8UG, UK; diane.ashiru-oredope@phe.gov.uk

**Keywords:** education, training, antimicrobial stewardship, behaviour change, quality improvement, workforce development

## Abstract

(1) Background: Pharmacists play a pivotal role in tackling Antimicrobial resistance through antimicrobial stewardship (AMS) and are well placed to lead behaviour change interventions across the healthcare system; (2) Methods: A cross-sector AMS training initiative for pharmacists was implemented across England, with three cohorts between 2019–2021. Each cohort took part in an introductory workshop, followed- by a workplace-based quality improvement project supported by peer-assisted learning sessions. Completion of training was determined by an end of training assessment after three to four months. Outcome data and learner survey results were collated, anonymised, and analysed by the training provider. (3) Results: In total, 118 pharmacists participated in the introductory workshop, 70% of these subsequently undertook an improvement project, and 48% engaged workplace stakeholders in the process. Interventions were designed by 57% of learners and 18% completed a at least one Plan-Do-Study-Act cycle. Approximately a quarter of learners met the requirements for a Certificate of Completion. Knowledge quiz scores were obtained from 115 learners pre-training and 28 learners post-training. Paired t-tests conducted for 28 learners showed a statistically significant improvement in mean score from 67.7% to 81.1% (*p* < 0.0001). Sixty-two learner survey responses were received during the training and 21 follow-up survey responses 6 to 12 months post training. Of the 21 responses to the follow-up survey, ongoing quality improvement work and improvement outcomes were reported by nine and six learners, respectively. (4) Conclusions: The delivery of workplace-based training at scale can be challenging, however this study demonstrates that coupling learning with workplace implementation and peer support can promote behaviour change in learners. Further study into the impact of providing pharmacists across sectors and geographies with access to this type of training will help inform ongoing workforce development interventions.

## 1. Introduction

It is estimated that antimicrobial-resistant infections cause approximately 700,000 deaths globally each year, a figure that may rise to 10 million by 2050 unless more is done to tackle this challenge locally and globally [1]. There have been efforts to harmonise interventions against antimicrobial resistance (AMR) in line with the World Health Organisation’s action plan published in 2015 [2]. In the UK, this has resulted in the development of a national 20-year vision, which is supported by action plans every five years [3,4,5].

As medication experts who are involved in all stages of patient management, pharmacists are well placed to influence antimicrobial prescribing and contribute to systemic efforts to curb AMR. In the UK, typical pharmacist roles and responsibilities include, but are not limited to, providing information and advice to patients, carers, and health professionals, supplying medicines and medical devices, patient consultations, as well as supporting education and training. The role of the pharmacist also varies between healthcare settings, with community pharmacists spending more of their time performing patient-facing and medication supply activities compared with those in secondary and primary care [6]. Conversely, pharmacists working in primary and secondary care likely to spend more time on providing information and advice to health professionals and medication reconciliation activities pre-/post-discharge [6].

Within the UK and internationally, pharmacist-led antimicrobial stewardship (AMS) activities have included guideline development and implementation, provision of clinical advice, audit and feedback, education, and management of IT systems [7,8]. In the National Health Service (NHS) within England, funding to secondary care organisations has been tied to national Commission for Quality and Innovation (CQUIN) indicators to drive improvements in prescribing. This provided an impetus for hospital trusts to invest in the development of their pharmacists to support the achievement of these key performance indicators. Whilst AMS activities in hospitals are primarily led by specialist antimicrobial pharmacists, generalist prescribing advisors are often responsible for stewardship in primary care [9,10]. There is a case for the broader involvement of pharmacists outside of these roles to lead AMS activities, including generalist clinical pharmacists and also those working in the community setting. The latter also play an important role in influencing how the general population utilise antimicrobials [11,12,13,14]. In England, regular monitoring of antimicrobial use and resistance has informed key priority areas across all sectors of healthcare, providing opportunities for pharmacists to support improvement [15]. 

Education and training have an important role in supporting the workforce to rise to the challenge, as evidenced by recent efforts to align AMS competencies between the professional curricula for medicine, nursing, pharmacy, and veterinary medicine [16,17,18]. However, the post-graduate training pathway for pharmacists in England is currently not standardised, with a variety of training delivered by workplaces, universities, and other providers in isolation. Although a national professional development roadmap and career stage milestones have been defined [19,20], there is still work being undertaken within the profession to provide a consistent approach to education and training, especially at the post-Foundation level. In 2014, an expert professional practice knowledge guide for antimicrobial pharmacists in the UK was published [21]. Although this was devised for antimicrobial pharmacists, it also highlighted areas that supported generalist practice [21]. In recent years, the availability of AMS resources for health professionals has expanded; however, the optimal use of education as a strategy to improve outcomes is yet to be established [22,23]. Furthermore, reviews of existing stewardship efforts have identified an underutilisation of behaviour change techniques, which could be integrated into professional curricula [22,24,25]. With the majority of training directed at medical practitioners, there is an opportunity to further support pharmacists to take a leadership role in AMS, especially as improvers and behaviour change champions [22,23]. This would align with key national policies, including the NHS Long-Term Plan and the UK AMR action plans [4,5,26,27,28]. 

In order to drive pharmacist-led AMS improvement, a publicly funded training course was delivered in England for postgraduate pharmacists from all sectors. The syllabus for this course covered introductory elements of AMS pharmacy practice [21], behaviour change, and quality improvement. Most importantly, learners were required to undertake an improvement project in the workplace. The syllabus aimed to facilitate learner capability, opportunity, and motivation to lead improvements [29], by utilising assessment and active learning strategies to influence their behaviour in the workplace. 

## 2. Materials and Methods

### 2.1. Funding, Training Design, and Delivery

The training was funded by Health Education England (HEE) through its AMR innovation fund. The development of the training syllabus was overseen by the Lead Pharmacist for Antimicrobial Resistance and Stewardship at the national public health institute (Public Health England). Learning activities were aligned to priorities outlined in the UK AMR five-year action plans [4,5]. Training content was identified from existing work on pharmacy professional development knowledge guides, aligned to Advanced Stage I practice as described in the nationally recognised Advanced Pharmacy Framework [20].

Three cohorts completed the training, which were each three to four months in duration. These consisted of a workshop and a workplace-based project component. Training was delivered in a blended format, with cohort 1 starting in March 2019, cohort 2 starting in February 2020, and cohort 3 starting in November 2020. Due to the COVID-19 pandemic, the training for cohort 2 was only partially delivered, and minor adjustments were made to the training for cohort 3. 

At the start of each cohort’s training, learners attended an introductory face-to-face workshop, with the exception of cohort 3, where this was delivered as two online webinars (see Supplementary Materials for workshop outline). The rest of the training was delivered virtually using a Learning Management System and video-conferencing technology. After the workshop, learners undertook self-directed learning against a combination of core and elective topics with the help of a study guide and curated online resources. All learners were required to demonstrate knowledge and understanding of core topics, which were deemed to be relevant across all sectors of pharmacy practice. Elective topics were curated to support setting-specific or more advanced practice, and learners were encouraged to identify those that were most relevant to their practice to be addressed in their individualised learning plans. All learners also undertook an AMS improvement project in their workplace on a topic of their choosing with the support of an improvement project guide and peer-assisted learning sessions in small groups. Each group consisted of up to six learners, supported by an allocated expert antimicrobial pharmacist tutor who facilitated four online discussion sessions. In these sessions, learners presented the progress of their projects and received structured feedback in a group setting.

Throughout the training, learners recorded reflective entries and collated evidence relating to their learning and improvement project. Together, these provided learners with a portfolio, which was used to demonstrate their achievement of the intended learning outcomes. Learners worked with their tutors and workplace stakeholders to establish improvement aims and measures. By the end of the training period, learners were required to demonstrate that a Plan-Do-Study-Act (PDSA) cycle test of change was either in progress or completed. 

An end of training assessment was scheduled for each cohort, which consisted of a one-on-one portfolio review and a virtual professional discussion with a consultant antimicrobial pharmacist or equivalent. Improvement project requirements, as well as the application of AMS and quality improvement knowledge and skills were assessed, using criteria aligned with expert professional practice and collaborative working competencies from the Advanced Pharmacy Framework [20]. Learners who demonstrated the required end of training outcomes were awarded a Certificate of Completion. Those requiring further support and/or time were provided with feedback as part of a professional development plan.

### 2.2. Learners, Tutors, and Assessors

Pharmacists practising in patient-facing or near-patient roles, who had at least 2 years of post-registration experience or completion of a postregistration Foundation Training programme were eligible to apply for a funded training place. Cohort 1 recruited 30 primary and secondary care pharmacists from the London and the South-east regions of England only. Cohort 2 and cohort 3 recruited 60 and 30 pharmacists, respectively, expanding eligibility criteria to include community pharmacists and all regions of England. HEE Pharmacy Deaneries and NHS employer networks were notified when enrolment was open in addition to advertisements on social media and email channels. Prospective learners applied online through an events management system. Learners’ applications were reviewed against the eligibility criteria prior to confirmation of their place on the training. Due to the finite number of funded places, eligible applications were accepted in the order that they were received. Applicants were discouraged from enrolling if they were undertaking concurrent training or academic study due to the risk of excessive workload. 

Nomination of a supervisor or manager to agree and support participation in the training was required as part of the application process. Workplace supervisors/managers were made aware of the requirement for learners to conduct an AMS improvement project during the training. The training was free of charge for learners with the exception of a small deposit, which was refunded on completion of the training. 

Tutors and assessors were recruited by disseminating expressions of interest via specialist antimicrobial pharmacist networks within the United Kingdom. Curricula vitaes were reviewed for prospective candidates in order to assess their suitability for the role, taking into consideration the balance of experience required to support learners from a diverse range of practice settings. Tutors and assessors received pro-rata financial remuneration for their participation in the training.

### 2.3. Data Collection and Evaluation

Demographic information, including years of practice, region, sector, and workplace, were collected from information submitted in the enrolment form. 

Learners’ knowledge of core topics was evaluated through a knowledge quiz completed pre-training and post-training. Quiz questions were validated by the training lead expert and a selection of external expert antimicrobial pharmacist tutors. In cohort 1, this multiple-choice question quiz comprised 30 questions. After further review and validation of the questions against the core learning topics, the quiz was updated and expanded to include 33 questions in cohorts 2 and 3. Each question was given a score of either 0 (incorrect), 0.5 (partially correct), or 1 (fully correct). After completing the quiz at the start of the training, learners were provided with their score and breakdown of results but were not provided with the correct answers. After completing the same quiz at the end of the training, learners were provided with the full set of answers in their results report. Access to the quiz was restricted on the Learning Management System to a single attempt each time, with access to the questions hidden between the pre- and post-training attempts. Scores were expressed as a percentage of the total score. The distribution of the pre- and post-training quiz results were analysed and compared. Where paired samples were available, a paired t-test was conducted to investigate whether the mean scores improved overall and for topic subgroups (AMS/infection and behaviour change/quality improvement). Comparisons were analysed using XLSTAT software (Data Analysis and Statistical Solution for Microsoft Excel^®^, Addinsoft, Paris, France 2017).

Materials submitted by learners, such as presentation slides, portfolio entries, and feedback forms, were collated to provide information on workplace AMS improvement activities. These were reviewed by a member of the training team who recorded and categorised the outcome data on Microsoft Excel^®^ for analysis. Learners were invited to complete a survey at the end of the training, which contained questions relating to their learning experience and perceived value of the training. The survey questions were reviewed and adapted between cohorts (see Supplementary Materials). In order to combine the analysis of these surveys, responses relating to learner experience were converted to a 5-point scale (1 = “very dissatisfied’ or ‘very poor’ and 5 = ‘very satisfied’ or ‘very good’) and analysed as a combined average score. Responses relating to the perceived value of the training were categorised as either positive, neutral, or negative. A follow-up survey was also sent out 6 to 12 months after the training, which contained questions relating to the ongoing continuation of the learner’s project and improvement practice in general.

### 2.4. Ethics Approval

Ethical approval was not required as per the NHS Health Research Authority guidance and the NHS health research decision tool [30]. Data collected for the evaluation of this project were anonymised. Participants were informed at the start of the training of the type and purpose of data collection for this review, and of their right to retract their anonymised data from the analysis at any time. No data pertaining to patients or other health service users were collected as part of this project. 

## 3. Results

### 3.1. Learner Demographics

**Figure 1:**
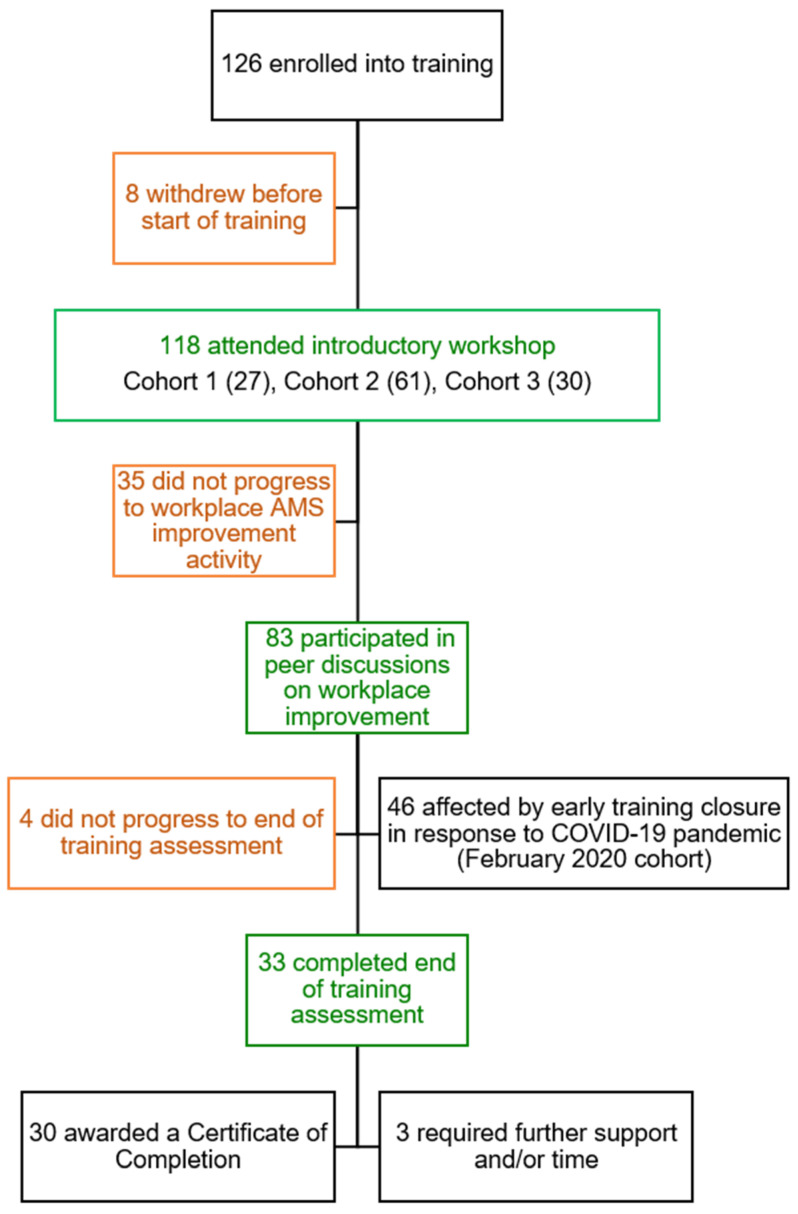
Summary of learner progression through training (2019–2021).

Figure 1 summarises learner progression across the three cohorts. Expressions of interest were received from 359 pharmacists in total, of which 126 were enrolled into the training. In total, 118 pharmacists attended the introductory workshop, of which 83 (70%) participated in peer discussion sessions on their workplace improvement initiative. Due to the COVID-19 pandemic, 46 learners (39%) were prevented from completing the training as cohort 2 closed early. Thirty-three learners (28%) progressed to the end of training assessment and 30 (25%) achieved a Certificate of Completion. As a percentage of learners from the cohorts where the training was delivered in full, this represents a completion rate of 53% (30 out of 57). 

The demographics of the learners who started and completed the training are outlined in Table 1. Of the 118 learners who attended the introductory workshops, 54% worked in acute care hospitals (secondary care), 35% worked in primary care, 8% in community pharmacy, and 3% in Community Health Trusts. Primary care learners included pharmacists employed by Clinical Commissioning Groups (CCGs), individual general practices (GP), or networks of GPs (Primary Care Networks). CCGs are independent statutory bodies that are responsible for commissioning primary care services and have a legal duty to support quality improvement. They are governed by members of local GPs with support from other health professionals, as well as patient and public representatives. Learners with a broad range of experience enrolled in the training, with 26% having practiced for less than 5 years, 36% having practiced for 5–10 years, and 38% having practiced for more than 10 years. Pharmacists worked in both clinical and non-clinical roles. Antimicrobial pharmacists comprised 19% of the learner demographic. 

Of the 85 learners who exited the training early after the introductory workshop, 41% occurred prior to the workplace-based project component and 59% occurred prior to the end of training assessment. In 30% of instances, the reason for this was unknown/not provided. The early closure of cohort 2 in response to the COVID-19 pandemic was the most common reason attributed to early exit from training, affecting 46 learners (54% of total early exits). Workplace-related factors were reported in 8% of early exits, such as disruptions to their usual role/team, or a lack of opportunity to conduct their AMS improvement project. Early exit from training was attributed to personal circumstances in 6% of cases and to workload in 2% of cases.

### 3.2. Knowledge Quiz

**Figure 2:**
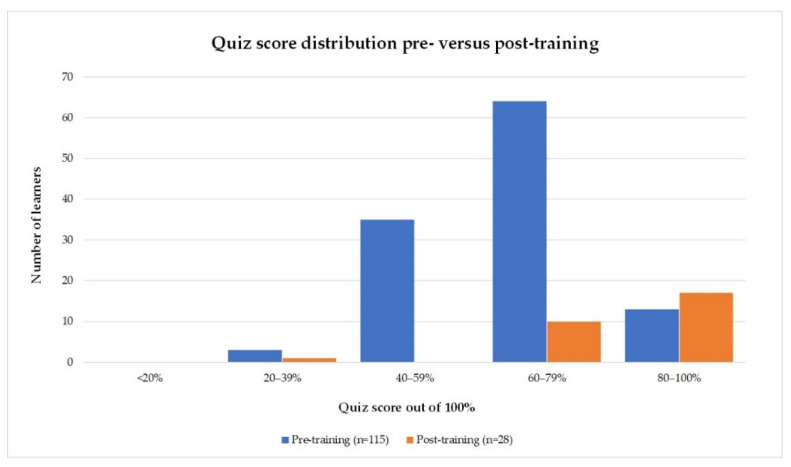
Comparison of quiz score distribution pre- versus post-training.

A total of 115 pre-training and 28 post-training knowledge quiz responses were analysed, representing 97% and 24% of the 118 learners who commenced the training, respectively. In cohort 1, there were 23 questions on AMS and infection management, and 7 questions on behaviour change/quality improvement. In subsequent quizzes, there were 26 questions on AMS and infection management, and 7 questions on behaviour change/quality improvement. The knowledge quiz questions are provided in the Supplementary Materials. Figure 2 illustrates the distribution of the pre- and post- training knowledge quiz score samples. In the pre-training sample, the majority of scores were between 60 and 79% compared with 80 and 100% in the post-training sample. 

Paired pre- versus post-training data was available for 28 learner quiz scores. The mean score increased from 67.7% to 81.1%. Paired t-tests showed that this was a statistically significant improvement in the mean total quiz scores as well as in the AMS/infection and behaviour change/quality improvement question sub-groups (Table 2).

### 3.3. Analysis of Learner Improvement Projects

**Figure 3:**
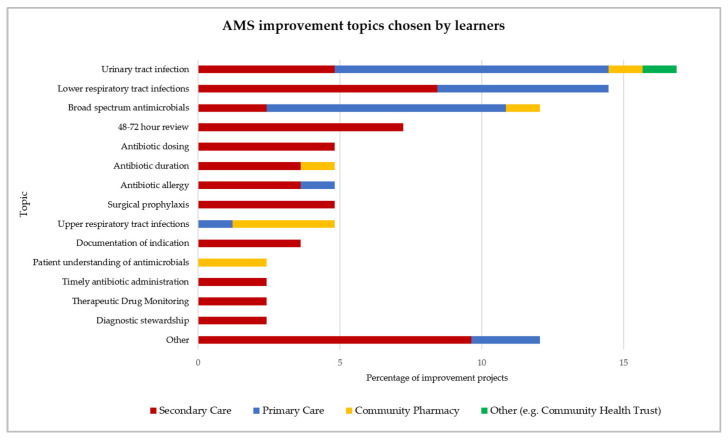
AMS improvement topics chosen by learners for their projects.

A total of 83 learners (70% of 118) identified an AMS topic to address in their workplace. Further details on these projects are provided in the Supplementary Materials. The most common improvement topic theme was urinary tract infection (17%), respiratory tract infections (14% lower and 5% upper), broad-spectrum antimicrobial prescribing (12%), and 48–72 h antimicrobial prescription review (7%) (Figure 3).

Table 3 outlines the activities undertaken by learners in the workplace as a part of their improvement projects. Approximately half of the learners (48% of 118) secured stakeholder engagement in the development of their project. These stakeholders were those who were directly impacted by the improvement project or were key to implementation. Examples included, but were not limited to, nursing staff, Infection Prevention and Control, AMS teams, and medical staff. Improvement interventions were devised by 67 of the learners (57% of 118), 25% collected data to improve the understanding of a problem, and 18% completed at least one PDSA cycle test of change. Thirty-one percent of learners promoted the improvement initiative outside of their team, for example, via bulletins or presentations, and 17% provided education to colleagues to support improvement.

Learners selected interventions targeting management prior to, at the time of, and post-prescription (Table 4). The implementation of a guideline or procedure was the most common pre-prescription intervention, whilst prospective audit and feedback was the most common post-prescription intervention. Interventions were most likely to address the Capability or Opportunity elements of the Behaviour Change Wheel [29], although in many cases they targeted multiple determinants of behaviour change. 

### 3.4. Learner Feedback and Follow-Up Surveys

Of the 118 learners who attended the introductory workshop, 40 withdrew their consent to be contacted by the end of the training. Invitations to complete a feedback survey were subsequently sent to the remaining 78 learners, of which 62 provided responses (10 from cohort 1, 39 from cohort, and 13 from cohort 3). This represents a response rate of 79% of surveyed learners, or 53% of total learners who started the training. When converting responses to learner satisfaction questions to a five-point scale, an average satisfaction score of 4.2 out of 5 was found. After categorising responses to questions relating to the perceived value of training as either positive, neutral, or negative, 90% provided a positive response, 10% provided a neutral response, and 0% provided a negative response. Further details on the survey questions and responses for the above are provided in the Supplementary Materials. 

A total of 21 learners responded to the follow-up survey at 6 to 12 months post training, yielding a response rate of 27% (21 of 78 surveyed learners) or 18% (21 of 118 total learners). From these responses, nine learners (43% of responses) reported that their improvement projects were continuing and six (29% of responses) reported having achieved improvement outcomes since the end of the training period. At the time of writing, the follow-up survey for cohort 3 was yet to be sent out and therefore was not included in this analysis. 

## 4. Discussion

To our knowledge, this is the first report of a freely accessible, cross-sector, workplace-based AMS training initiative for pharmacists that has been implemented at a national level. By involving pharmacists from a diverse range of workplaces, roles, and levels of experience, this training embodies the provision of education across sectors and at all levels (“from board to floor”), as outlined in the UK’s 20-year vision for AMR [3]. The incorporation of peer-assisted learning also encourages collaboration and sharing of knowledge, fostering the development of systems leadership across geographic, speciality, and workplace boundaries. The involvement of expert antimicrobial pharmacists as tutors and assessors further enhances their existing roles as leaders, mentors, and advocates. Pharmacists currently have limited opportunities to develop their quality improvement and behaviour change skills. Furthermore, the training that post-graduate pharmacists receive varies across workplaces and healthcare settings. The training model we have described could provide a consistent approach to introducing pharmacists to these important topics, as well as supporting them to apply these skills, especially if expertise is not available within the workplace. In the future, this training could be used to support all pharmacists to develop and demonstrate a subset of the required credentialing competencies within a standardised pathway.

Across the three cohorts delivered to date, learner demand has far exceeded the available number of funded places, demonstrating the appeal of this training to the profession. Although the training was not aimed at a specialist audience or pharmacists practicing beyond Advanced Stage II of the Advanced Pharmacy Framework [20], the majority of pharmacists had practiced for more than five years, with 38% having practiced for more than 10 years. More than 80% of learners were generalist pharmacists, which was the primary intended audience of the training. The diversity of patient-facing and non-patient-facing roles was also reflected in the cohorts, including clinical pharmacists, antimicrobial pharmacists, prescribing advisors, pharmacy managers, medicines optimisation pharmacists, and community pharmacists. A survey in 2016 found that 5% of CCGs had antimicrobial pharmacists, compared to 83% of acute trusts in secondary care [10]. In our cohorts, antimicrobial pharmacists made up almost 20% of the learner cohorts, despite the curriculum being aimed at generalists. As the majority of these pharmacists were new to their posts, the training supported their transition into their new roles, in the absence of a standardised training pathway. One of the benefits of having heterogenous cohorts in terms of experience, roles, and workplace settings was that it encouraged systems leadership. However, this also limited the extent to which the training could be individualised. In our training delivery, this was mitigated through the inclusion of elective topics that allowed learning plans to be tailored based on roles and level of experience. Learner backgrounds were also taken into account when allocating tutors, assessors, and peer group members.

Primary and secondary care pharmacists had a higher level of representation compared with community pharmacists, with the latter making up 8% of the learner cohorts despite making up 63% of the pharmacy workforce according to a recent survey [6]. Although part of the wider primary care team, community pharmacists may face barriers unique to their working environment, such as different levels of expectations and support for quality improvement training [31]. Targeted collaboration with community pharmacy bodies can assist in improving employer buy-in and learner recruitment, as demonstrated in a recent initiative to provide training on consultation skills to community pharmacists across England [32]. The showcasing of work from previous community pharmacy pharmacist learners would also raise awareness and promote the value of training amongst colleagues. 

It was hypothesised that this training would influence learner behaviours, supporting their capability, opportunity, and motivation to lead small-scale AMS improvement in their workplace [29]. Capability was developed through the introductory workshop, tutor feedback, and directed self-learning. The improvements in quiz scores suggest that the training was successful in improving knowledge and understanding of core AMS and behaviour change/quality improvement principles, with this improvement being statistically significant in learners who completed the training in full. Social opportunity, as defined by interpersonal influences, social cues, and cultural norms, was fostered through the use of synchronous, small group sessions at scheduled milestones, where learners presented and discussed their progress. Motivation, such as goal setting and anticipation of a reward, was influenced through the end of training portfolio review and Certificate of Completion requirements, as an example of using assessment drive behaviour [29]. 

Due to the number of learners who exited early from the training, largely driven by the impact of the COVID-19 pandemic, it was difficult to evaluate the full impact of the training when undertaken to completion. The proportion of learners who completed all of the required training activities was 28%, with 25% demonstrating the required outcomes at the end of training assessment. The high levels of satisfaction and perceived value reported in the learner surveys need to be interpreted in the context of a low response rate (53%), and a potential bias towards those who were more engaged with the training. The majority of respondents did not complete the training in full, hence the results likely reflect learner views on the training workshop and peer discussion sessions only rather than all of the training activities. The proportion of positive responses remained high despite 39 of the 62 respondents (63%) being affected by the early closure of their training due to COVID-19.

The early closure of cohort 2 was a significant contributor to the low completion rate, with illness and workplace disruptions, such as redeployment to the delivery of COVID-19 clinical care, being key factors behind this decision. The majority of the other early learner exits from training occurred prior to the workplace-based AMS improvement component of the training course. This suggests that learners may find the initiation stages of AMS improvement particularly challenging and that they would benefit from additional engagement and support in developing their projects. 

The three to fourth month training duration was shorter than that of other programmes, where learners were required to conduct quality improvement in the workplace [33,34]. This was because the training aimed to provide an introduction to quality improvement/AMS whilst encouraging new learner behaviours, rather than to assess completion of a quality improvement project in its entirety. Other considerations for this included the logistics of training provision at a national level and feasibility for learners, tutors, and assessors to commit to training activities over a longer time frame. Nonetheless, the short duration of training is likely to have been a barrier to training completion, especially for learners who are attempting to instigate significant practice changes and those with limited research expertise or workplace support. Strategies to facilitate further support in the workplace would be beneficial, although challenging to implement logistically at scale. In line with intended application of PDSA in the Model for Improvement, the short training duration also aimed to encourage learners to implement iterative, rapid tests of change. However, this was a concept that many learners struggled with, resulting in only 19% of learners completing a test of change during the training. Suggested strategies to improve uptake and correct application have included staggering the teaching activities, to focus on learner perceptions and attitudes to PDSA cycles at the start of the training, followed by more detailed information on implementation later on to provide ‘just in time’ learning [33].

Despite these limitations, there was evidence that the training resulted in learners undertaking meaningful activities in the workplace, including by those who exited the training early. Almost half (48%) had engaged workplace stakeholders or had designed an intervention to address their area of improvement (57%). The level of collaboration with workplace stakeholders is encouraging as this is fundamental to the achievement of quality improvement and behaviour change. There was also evidence of promotional activities, education provision, and exploratory data collection to improve understanding of a problem, which are also valuable outcomes. It was encouraging to find examples of improvement projects continuing 6 to 12 months post training (8% of learners), and of ongoing improvement outcomes reported by learners (5% of learners). Further evaluation is recommended to better understand these long-term impacts, given the small response rate and limited assessment of outcomes undertaken in the follow-up survey. A lack of incentive for learners to report follow-up data poses a barrier to the evaluation of the training’s impact. Strategies to secure commitments from learners and/or employers to provide follow-up data should be considered in the future as part of the enrolment process.

Improvement topics, which were chosen at the discretion of each learner, were aligned with national AMS/AMR priorities, such as those described by CQUIN indicators and Antibiotic Quality Premiums. Common examples included urinary tract infection (17%), broad-spectrum antibiotic prescribing (12%), as well as antibiotic review, surgical prophylaxis, and time to antibiotics in sepsis [35,36,37]. Learners were also responsive to local priorities outside of the published national indicators, in areas, such as Therapeutic Drug Monitoring, diagnostic stewardship, and antibiotic allergy documentation. 

Learners were most likely to choose intervention designs that were commonly reported in the AMS literature, such as implementing guidelines/procedures, audit with feedback, and practice aids. These interventions were often coupled with other engagement strategies, such as promotion and education. Despite common themes in the choice of interventions, there was variation in how these were implemented and therefore the behaviour change determinants that were addressed. For example, some learners influenced social opportunity or reflective motivation more than others in their approach to conducting audit and feedback, or when implementing a guideline. The preference for the use of guidelines and audit with feedback is likely due to improved familiarity and perceived ease of implementation, especially given the time constraints of the training and proportion of learners from the hospital setting. The fact that most learners were new to quality improvement and developing behaviour change interventions may have also been a factor. There is a need to test and implement a broader range of behaviour change interventions; therefore, strategies to encourage this would be beneficial in the future [25]. 

A number of human, logistical, and technological resources were required to deliver this training. This included Learning Management System infrastructure, and expertise in delivering remote learning, including the capacity to co-ordinate and regularly engage large cohorts of participants. This was especially pertinent given the wide spread of learners, tutors, and assessors across organisations, settings, and geography. Co-ordinating the availability of all those involved in the training was a logistical challenge, influenced by variable working patterns and co-existing time commitments. This required a flexible approach to training delivery. Despite these challenges, the outcomes of this study demonstrate that there are tangible benefits to using blended workplace-based training to promote AMS behaviour change in the workforce. This study also reinforces the benefits of incorporating real-world problems and the provision of mentoring with feedback over didactic teaching alone, further building upon the limited but growing evidence base in this area [34]. 

## 5. Conclusions

This study provides a real-life example of how a national cross-sector training initiative can be delivered to promote pharmacist-led AMS improvement aligned to national priorities. It is also a case study of how behaviour change methodology can be integrated with education design principles, such as active learning and the use of assessment to drive learner behaviour. 

It is difficult to extrapolate the improvements in knowledge and learner feedback to the broader learner population due to the low completion rate in this study. Further evaluation of the training outside of the pandemic context would be informative, especially in terms of optimal delivery and impact outcomes. Although COVID-19 was likely the most significant contributor to the low completion rate, attention should also be paid to learner understanding of the correct use of PDSA in quality improvement, training duration, and workplace-based barriers to training completion. 

Despite the low proportion of completed PDSA cycles and end of training assessments, many learners were still able complete valuable preliminary quality improvement activities in the workplace, which supports the hypothesis that the training can influence behaviour. Future development of this training initiative could include expansion to a multi-disciplinary model, as well as integration into a standardised pharmacy training pathway.

## Figures and Tables

**Table 1:** Summary of learner demographic information.

Number of Learners (% of Sub-Group)		
Demographic	Started the Training	Certificate of Completion
Region		
North	15 (12%)	5 (16%)
Midlands	15 (13%)	2 (7%)
East	16 (14%)	2 (7%)
London	36 (30%)	9 (30%)
Southwest	8 (7%)	0 (0%)
Southeast	28 (24%)	12 (40%)
Sector		
Hospital	64 (54%)	19 (63%)
CCG	25 (21%)	5 (16%)
GP/PCN	16 (14%)	2 (7%)
Community pharmacy	10 (8%)	2 (7%)
Community health trust	3 (3%)	2 (7%)
Years of experience		
<5 years	31 (26%)	9 (30%)
5–10 years	42 (36%)	9 (30%)
>10 years	45 (38%)	12 (40%)
Workplace roles		
Clinical pharmacist	62 (53%)	18 (60%)
Antimicrobial pharmacist	23 (19%)	4 (13%)
Prescribing advisor	11 (9%)	2 (7%)
Pharmacy manager	9 (8%)	3 (10%)
Medicines optimisation	9 (8%)	3 (10%)
Community pharmacist	4 (3%)	0 (0%)
Total	118 (100%)	30 (100%)

**Table 2:** Comparison of paired learner knowledge quiz data pre- versus post-training (*n* = 28).

Quiz Section	Sample	Mean Score (%)	SD	*t*	*p*-Value
All questions	pre	67.7	10.4	−5.43	<0.0001
	post	81.1	14.4		
AMS and infection management	pre	65.0	10.3	−6.09	<0.0001
	post	79.4	14.4		
Quality improvement and behaviour change	pre	76.8	19.5	−2.26	0.03
	post	86.7	19.3		

**Table 3:** Activities undertaken by learners in the workplace as part of their improvement projects.

Activity	Number of Learners			
	Cohort 1	Cohort 2	Cohort 3	Total (% of 118 Learners)
Identified improvement topic	19 (16%)	45 (38%)	19 (16%)	83 (70%)
Secured stakeholder engagement	19 (16%)	25 (21%)	13 (11%)	57 (48%)
Improved understanding of problem through data collection	10 (9%)	4 (3%)	15 (13%)	29 (25%)
Devised an improvement intervention	19 (16%)	34 (29%)	14 (12%)	67 (57%)
Conducted a test of change	17 (14%)	0 (0%)	5 (4%)	22 (18%)
Promoted improvement initiative	15 (13%)	9 (8%)	12 (10%)	36 (31%)
Provided education to colleagues	10 (8%)	1 (1%)	9 (8%)	20 (17%)

**Table 4:** Improvement interventions devised by learners during the training.

Characteristics of Interventions	Number of Interventions (% of Interventions)	PDSA Cycle Completed (% of Interventions)
Proposed intervention type:		
Guideline or procedure	14 (20%)	4 (6%)
Prospective audit and feedback	11 (16%)	4 (6%)
Use of practice aids	12 (18%)	4 (6%)
Education and promotion	13 (19%)	3 (4%)
Prompts and reminders	8 (12%)	2 (3%)
Restriction	5 (7%)	3 (4%)
Electronic prescribing functions	1 (2%)	1 (2%)
Role modelling	1 (2%)	1 (2%)
Peer group benchmarking	1 (2%)	0 (0%)
Clinical decision support	1 (2%)	0 (0%)
Behavioural determinants addressed:		
Capability	24 (36%)	5 (7%)
Opportunity	19 (28%)	9 (13%)
Motivation	9 (13%)	2 (3%)
Combination of capability, opportunity and/or motivation	15 (23%)	6 (10%)
Total number of interventions	67 (100%)	22 (33%)

## Data Availability

The data presented in this study are available on request from the corresponding author. The data are not publicly available due to privacy.

## Supplementary Materials

The following are available online at www.mdpi.com/article/10.3390/pharmacy9040165/s1, File S1: Introductory workshop outline, File S2: Learner survey questions, File S3: Knowledge quiz (cohort 1), File S4: Knowledge quiz (cohorts 2 and 3), File S5: Learner improvement project activities, File S6: Learner feedback survey responses (learner experience and value of training).
