# Supplementary material for: Outcomes of a National, Cross-Sector Antimicrobial Stewardship Training Initiative for Pharmacists in England
Source: Pharmacy (Basel). 2021 Oct 10;9(4):165. doi: 10.3390/pharmacy9040165 (PMC8544733; doi:10.3390/pharmacy9040165)
Supplement: Supplementary file 1 [file pharmacy-09-00165-s001.zip › Supplementary materials (S1) - introductory workshop outline.pdf]

ROYAL
PHARMACEUTICAL
SOCIETY

Antimicrobial Stewardship Training Day Programme

Time	Session
10:00 – 10:30	Registration, tea and networking
10:30 – 11:00	Welcome and introductions
11:00 – 11:40	Tackling AMR through AMS interventions
11:40 – 12:40	Using AMR/AMS Data for Action
12:40 – 13:20	Lunch and networking
13:20 – 15:00	Designing Antimicrobial Stewardship interventions using behaviour change theory
15:00 – 15:15	Refreshments
15:15 – 17:15	Applying quality improvement to Antimicrobial Stewardship
17:15 – 17:30	Feedback surveys, action planning, and next steps
