# Supplementary material for: Outcomes of a National, Cross-Sector Antimicrobial Stewardship Training Initiative for Pharmacists in England
Source: Pharmacy (Basel). 2021 Oct 10;9(4):165. doi: 10.3390/pharmacy9040165 (PMC8544733; doi:10.3390/pharmacy9040165)
Supplement: Supplementary file 1 [file pharmacy-09-00165-s001.zip › Supplementary materials (S2) - learner survey questions.pdf]

Cohort 1 learner feedback survey questions

RPS AMS Training Programme Feedback form

Thank you for all your hard work in the last few months!

Help us improve this training for future participants by answering this short survey. This is an anonymous survey, however if you would like to discuss your feedback further with us, you can contact us via education@rpharms.com.

Required

1. Give us a brief summary of your thoughts on the training and any impacts on your practice.

   Enter your answer

2. Rate your overall experience of the training programme

   Very dissatisfied … Very satisfied

3. Would you recommend this training to a colleague?
   - Yes
   - No
   - Maybe

4. Select the option that best describes your thoughts on each component of the training

	Not useful	Would need significant change to be beneficial	Could have been beneficial if improved	Beneficial, but could be improved	Beneficial, I wouldn't change it
End of training assessment	○	○	○	○	○
Knowledge + Capability Guide	○	○	○	○	○
Online feedback sessions	○	○	○	○	○
CPD entries - peer discussion entry and planned learning entries	○	○	○	○	○
CPD entry - reflective account	○	○	○	○	○
Moodle website	○	○	○	○	○
Training Day	○	○	○	○	○
Self-directed learning resources	○	○	○	○	○
End of training assessment	○	○	○	○	○
Quality Improvement toolkit	○	○	○	○	○
APF mapping matrix	○	○	○	○	○

5. Based on your response above, what would you most like to see improved and how?

Enter your answer

6. What are your thoughts on adding a second face to face training day?

- This would be of value and would be feasible to commit to
- This would be of value buy would NOT be feasible to commit to
- One training day is enough
- I would prefer self-directed learning or interactive online learning over face to face training days

7. What would be your preferred avenue to ask questions relating to the training?

- Moodle forums
- Email
- Other

8. Tell us your thoughts on the use of Moodle forums and discussion boards.

Enter your answer

9. Rate the quality of ythe teaching and feedback provided by your tutor(s)

- Very poor
- Poor
- Average
- Good
- Very good

10. Rate the quality of the feedback provided by your end of training assessor

- Very poor
- Poor
- Average
- Good
- Very good

11. Rate the quality of the support that you received from the RPS professional development team during the training

- Very poor
- Poor
- Average
- Good
- Very good

12. Provide more information or additional comments relating to your ratings for the two previous questions

Enter your answer

13. Was the workload manageable?

- Yes
- No

14. Approximately how many hours a week OUTSIDE of work hours did you spend on this training programme, EXCLUDING assessments, skype sessions and the training day

- <2 hours a week
- 2-3 hours a week
- 4-6 hours a week
- > 6 hours a week

15. How much would you be willing to pay for this training?

- ◯ I wouldn't pay for this training
- ◯ <£100
- ◯ £100-300
- ◯ £300-500
- ◯ >£500

16. What is the likelihood of you becoming a RPS member or renewing your RPS membership since completing this training?

- ◯ More likely
- ◯ Neither more or less likely
- ◯ Less likely
- ◯ Unsure

17. What would you like to see from us in terms of education and training in order to increase the likelihood of you joining the RPS or renewing your membership?

Enter your answer

18. What are the biggest barriers to your Professional Development in terms of education and training?

Cohort 2 learner feedback survey questions

1 *

Give us a brief summary of your thoughts on the training and any impacts on your practice.

2 *

Rate your overall experience of the training programme.

- ○ Very satisfied
- ○ Satisfied
- ○ Neither satisfied or dissatisfied
- ○ Dissatisfied
- ○ Very dissatisfied

3 *

Would you recommend this training to a colleague?

- ○ Yes
- ○ No
- ○ Maybe

4

Provide further information on your response to the previous question:

5 *

Rate your current level of knowledge in the following areas:

	Very low	Low	Average	High	Very high
1. The 7 key areas identified nationally to tackle AMR	○	○	○	○	○
2. All the visualisation tools available for antibiotic prescribing in primary care (e.g. PHE Fingertips, PrescQIPP AMS and data hubs, openprescribing.net, ePact2)	○	○	○	○	○
3. All the visualisation tools available for antibiotic prescribing in secondary care (e.g. PHE Fingertips, RxInfo Define, NHS Model Hospital dashboard)	○	○	○	○	○
4. Quality improvement methodology and tools	○	○	○	○	○
5. How to design AMS interventions using behaviour change techniques	○	○	○	○	○

6 *

Rate your current level of involvement in the following activities:

	Rarely	Sometimes	Usually	Consistently	N/A
1. Review and optimisation of antimicrobial prescriptions (indication, dose and duration)	○	○	○	○	○
2. Review and optimisation long-term/repeat antimicrobial prescription prescribing	○	○	○	○	○
3. Contribute to or lead a formal AMS programme in your workplace	○	○	○	○	○
4. Promote AMS to colleagues through education or training	○	○	○	○	○
5. Use of data within AMS practice to improve patient outcomes and safety	○	○	○	○	○
6. Contribute to projects that implement change to improve Antimicrobial Stewardship practices	○	○	○	○	○
7. Lead projects that implement change to improve Antimicrobial Stewardship practices	○	○	○	○	○

7 *

How beneficial were the following training activities/resources to your learning/development?

	Not beneficial at all	Not very beneficial	Neutral	Beneficial	Very beneficial
Online moodle activities (e.g. AMS in your workplace)	○	○	○	○	○
Knowledge quiz	○	○	○	○	○
Group discussion sessions with tutor	○	○	○	○	○
CPD entries	○	○	○	○	○
QI workbook	○	○	○	○	○
Training Day	○	○	○	○	○
Self-directed learning resources	○	○	○	○	○

8 *

Rate the quality of the following:

	Very poor	Poor	Average	Good	Very good
Online learning resources	○	○	○	○	○
Tutor teaching and feedback	○	○	○	○	○
RPS support	○	○	○	○	○

9 *

Provide further information or additional comments relating to your ratings for the two previous questions.

10 *

Which of the following channels best describe your interactions with your group and tutor outside of the online sessions:

- ☐ Email
- ☐ Whatsapp/telegram group
- ☐ Teleconferencing
- ☐ In person
- ☐ Other (please specify): ____
- ☐ Not applicable

11 *

If the COVID-19 pandemic was not a factor, how manageable would the workload have been?

- ○ Very manageable
- ○ Manageable
- ○ Neutral
- ○ Not very manageable
- ○ Not manageable at all

12 *

If the programme was to be delivered entirely remotely, how would you like to see the face-to-face day incorporated into a digital/online format?

13 *

What areas of your work or career would you like to be better supported in? How would you like to be supported?

14

If you have any further comments or feedback, please provide it here:

Cohort 3 learner feedback survey questions

1 *

Rate the quality of the following aspects of the training programme:

	Very poor	Poor	Average	Good	Very good	Not applicable
Learning resources	○	○	○	○	○	○
Learning activities	○	○	○	○	○	○
Communication and support from the RPS	○	○	○	○	○	○
Tutor support and feedback	○	○	○	○	○	○
Assessors support and feedback	○	○	○	○	○	○
Planning and organisation	○	○	○	○	○	○
The learning environment	○	○	○	○	○	○
Overall training experience	○	○	○	○	○	○

2 *

Select the rating that best matches your thoughts on the following statements about the training.

	Strongly disagree	Disagree	Neither agree or disagree	Agree	Strongly agree
The learning outcomes were relevant and appropriate for my practice.	○	○	○	○	○
The learning activities were effective at helping me develop the required knowledge and skills.	○	○	○	○	○
The balance between self-directed learning and instructor-lead learning was optimal.	○	○	○	○	○
The number and frequency of learning activities was optimal.	○	○	○	○	○
Participating in this training was a valuable use of my time.	○	○	○	○	○

3

Based on your responses for the previous 2 questions, please tell us about anything particularly **GOOD** or **BAD** about the training here:

4

Please provide one suggestion to improve this course.

5

What topics would you be be interested in seeing covered by future RPS Education products and services, such as training, webinars, and resources?

6 *

Would you be happy to be contacted by the RPS in the future for the following?

	Yes	No
Provide a testimonial about the training	○	○
Invitation to showcase your work	○	○
Follow up survey 6-12 months after the end of the training	○	○

Follow-up survey questions (6-12 months post-training)

1. What was your training programme project?

   [free text]

2. Which of the following options best describes your involvement in AMS since completing the training?

"I have planned, managed, monitored, advised and reviewed pharmaceutical care programmes in the area of AMS"

- Rarely or not applicable
- Sometimes
- Usually
- Consistently
- I have provided in-depth or complex AMS care

3. Which of the following best describes your involvement in QI before enrolling into the training?

- None
- I had a minor role in someone else's project – e.g. consulted as a stakeholder
- I was actively collecting data or implementing interventions under the direction of someone else
- I was leading or making significant contributions to the planning and design of QI regularly (at least one project a year)

5. Is your AMS training QI project still continuing?

- Yes
- No

6. What is your current involvement in QI? [Can select more than 1]

- I am still leading my AMS training QI project
- I have handed over my AMS training QI project to someone else
- I am contributing to a different QI project led by someone else
- I am leading a different QI project
- I am not involved in any quality improvement activity anymore

Page Break

7. Which of the following best describe the stage of your AMS training QI project **during the training program**?

- Planning
- Proof of concept – you explored the effects of a novel or bespoke intervention on a very small scale
- Testing – you took an intervention that has been trialled before and refined it for your setting
- Spread and scale up – you took an intervention that has been tested in your setting and expanded its implementation to the wider organisation or region

8. Which of the following best describe the stage of your AMS training QI project **since the end of training**?

- Planning
- Proof of concept – you explored the effects of a novel or bespoke intervention on a very small scale

- Testing – you took an intervention that has been trialled before and refined it for your setting
- Spread and scale up – you took an intervention that has been tested in your setting and expanded its implementation to the wider organisation or region

9. In relation to your AMS QI initiative, which of the following have been demonstrated **since the end of training**? [can select more than 1]

- Improved care processes and practices
- Improved patient outcomes and experience of care
- Increased efficiencies (including cost) and standardisation
- Improved staff satisfaction or knowledge
- Enhanced culture of quality and improvement
- Behaviour change
- Other
- None of the above – skip to question 11

10. How have you determined your evaluation outcome(s) from question 8 [can select more than 1]?

- Consensus within your project team/stakeholders
- A run chart analysis with a minimum of 6 data points
- Pre- and post- intervention audits with representative sample sizes (i.e. using a sample size calculator, external guidelines or other scientific method)
- Anecdotal

11. Have you shared your QI work since the end of training [can select more than 1]

- Yes –poster or oral presentation at conference
- Yes – within your department or project team
- Yes – to those within your organisation but outside of my department or project team
- Yes – to those outside of your organisation
- No

12. Feel free to provide any further information or details on the progress of your QI initiative here:
