# Supplementary material for: Outcomes of a National, Cross-Sector Antimicrobial Stewardship Training Initiative for Pharmacists in England
Source: Pharmacy (Basel). 2021 Oct 10;9(4):165. doi: 10.3390/pharmacy9040165 (PMC8544733; doi:10.3390/pharmacy9040165)
Supplement: Supplementary file 1 [file pharmacy-09-00165-s001.zip › Supplementary materials (S3) - Knowledge quiz (cohort 1).pdf]

Question 1
Not complete
Weight 1.00

Which of the following are mechanisms by which organisms can transfer resistance genes? **(Select all that apply)**

Select one or more:

- a. Transcription
- b. Conjugation
- c. Transduction
- d. Transformation
- e. Budding

Certainty : C=1 (Unsure: <67%) C=2 (Mid: >67%) C=3 (Quite sure: >80%)

Check

Question 2
Not complete
Weight 1.00

In January 2019, the UK launched a new 5-year action plan to tackle Antimicrobial Resistance (AMR) alongside a 20 year vision for AMR. Which of the following statements reflect the proposed plans and intentions of the action plan and vision? **(Select all that apply)**

Select one or more:

- a. AMR should be contained and controlled
- b. The UK supports a targeted approach to the AMR problem with efforts focused on supporting countries with high antibiotic use such as India and the farming industry in the UK
- c. Intentional antibiotic use should be reduced
- d. The UK is committed to a local, national and global approach across humans, animals, the environment and food in order to sustain efforts in addressing the AMR problem.
- e. Antibiotic use and unintentional exposure to antibiotics should be reduced.
- f. AMR should be reduced and revised

Certainty : C=1 (Unsure: <67%) C=2 (Mid: >67%) C=3 (Quite sure: >80%)

Check

Question 3
Not complete
Weight 1.00

Which of the following tools are openly accessible? **(Select all that apply)**

Select one or more:

- a. Rx-Info Define
- b. OpenPrescribing.net
- c. PrescQIPP Data Hub
- d. ePACT2
- e. PHE Fingertips
- f. PrescQIPP Antimicrobial Stewardship Hub
- g. NHS Model Hospital

Certainty : C=1 (Unsure: <67%) C=2 (Mid: >67%) C=3 (Quite sure: >80%)

Check

Question **4**

Not complete

Weight 1.00

Which of the following tools mainly uses primary care reimbursement data to illustrate prescribing? **(Select all that apply)**

Select one or more:

- [ ] a. NHS Model Hospital
- [ ] b. PHE Fingertip
- [ ] c. OpenPrescribing.net
- [ ] d. PrescQIPP Antimicrobial Stewardship Hub
- [ ] e. PrescQIPP Data Hub
- [ ] f. ePACT2
- [ ] g. Rx-Info Define

Certainty ⓘ : ○ C=1 (Unsure: <67%) ○ C=2 (Mid: >67%) ○ C=3 (Quite sure: >80%)

Check

Question **5**

Not complete

Weight 1.00

When should you dip a urinary catheter?

Select one:

○ a. When you have an unclear diagnosis and suspect UTI

○ b. When you have no other source of reliable urine

○ c. Never

Certainty ⓘ : ○ C=1 (Unsure: <67%) ○ C=2 (Mid: >67%) ○ C=3 (Quite sure: >80%)

Check

Question **6**

Not complete

Weight 1.00

Why are urinary catheters problematic for UTI?

Select one:

○ a. A biofilm can be created which harbours microbes antibiotics cannot reach

○ b. Stagnant urine in situ

○ c. External source of infection from outside the body

Certainty ⓘ : ○ C=1 (Unsure: <67%) ○ C=2 (Mid: >67%) ○ C=3 (Quite sure: >80%)

Check

Question **7**

Not complete

Weight 1.00

Choose the most appropriate course of action for the following condition. Patient with leg ulcer. Cultures have grown Pseudomonas + MRSA

Select one:

○ a. Treat with oral linezolid

○ b. Treat with IV Ciprofloxacin +Vancomycin/Teicoplanin

○ c. No antibiotic treatment unless there is surrounding cellulitis

○ d. Treat with oral vancomycin

Certainty ⓘ : ○ C=1 (Unsure: <67%) ○ C=2 (Mid: >67%) ○ C=3 (Quite sure: >80%)

Check

Question **8**

Not complete

Weight 1.00

Choose most appropriate course of action. You see a patient with blocked urinary catheter, urine cultures have grown > 10^5 CFU/mL *Pseudomonas* species.

Select one:

○ a. IV ceftazidime

○ b. Oral ciprofloxacin

○ c. Co-amoxiclav if there are signs of infection

○ d. No antibiotic therapy unless there are signs of systemic sepsis

Certainty ⓘ : ○ C=1 (Unsure: <67%) ○ C=2 (Mid: >67%) ○ C=3 (Quite sure: >80%)

Check

Question 9
Not complete
Weight 1.00

According to the NICE guideline for acute cough which of the following antibiotics is the **preferred first-line agent**, if prescribed for adults who are systemically unwell or at risk of complications.

Select one:

- a. amoxicillin
- b. cephalexin
- c. doxycycline
- d. clarithromycin

Certainty ? : C=1 (Unsure: <67%) C=2 (Mid: >67%) C=3 (Quite sure: >80%)

Check

Question 10
Not complete
Weight 1.00

Asymptomatic bacteriuria should always be treated in:

Select one:

- a. Pregnant women
- b. All of the above
- c. Children
- d. Elderly

Certainty ? : C=1 (Unsure: <67%) C=2 (Mid: >67%) C=3 (Quite sure: >80%)

Check

Question 11
Not complete
Weight 1.00

Which of the following are risk factors for *Clostridium difficile* infection? **(Select all that apply)**

Select one or more:

- a. Broad spectrum antibiotics
- b. Transmission from another source
- c. Proton pump inhibitors

Certainty ? : C=1 (Unsure: <67%) C=2 (Mid: >67%) C=3 (Quite sure: >80%)

Check

Question 12
Not complete
Weight 1.00

Which age group is more likely to develop E. coli bacteraemia?

Select one:

- a. <65 years
- b. >80 years
- c. 65-80 years

Certainty ? : C=1 (Unsure: <67%) C=2 (Mid: >67%) C=3 (Quite sure: >80%)

Check

Question 13
Not complete
Weight 1.00

The following are reliable markers of infection: **(Select all that apply)**

Select one or more:

- a. White Blood Cells
- b. Glasgow Coma Score
- c. Body temperature
- d. Procalcitonin
- e. Haemoglobin
- f. CRP
- g. Neutrophils

Certainty ? : C=1 (Unsure: <67%) C=2 (Mid: >67%) C=3 (Quite sure: >80%)

Check

Question **14**

Not complete

Weight 1.00

Which of the following is **false**?

Select one:

- a. Hospital acquire pneumonia can be prevented by getting patients sat out if bed or upright
- b. Giving IV antibiotics to patients while in hospital reduces risk of HCAI
- c. C.difficile is classified as a healthcare associated infection

Certainty : C=1 (Unsure: <67%) C=2 (Mid: >67%) C=3 (Quite sure: >80%)

Check

Question **15**

Not complete

Weight 1.00

Mr T has recently been discharged from hospital. He was diagnosed with Tuberculosis and started on medication, Rifater (rifampicin, isoniazid and pyrazinamide) with ethambutol. Two weeks later he reports that he notices that his skin colouration and eyes have become slightly yellow and he has been very tired lately.

Select one:

- a. He should stop taking ethambutol straight away and see his GP as soon as possible
- b. The symptoms described are not known to be caused by Rifater or ethambutol.
- c. He should stop taking Rifater straight away and see her GP as soon as possible
- d. He is experiencing a side-effect of Rifater, and whilst safe to continue taking it, he may wish to see his GP for an alternative
- e. He experiencing a side-effect of ethambutol, and whilst safe to continue taking it, he may wish to see his GP for an alternative

Certainty : C=1 (Unsure: <67%) C=2 (Mid: >67%) C=3 (Quite sure: >80%)

Check

Question **16**

Not complete

Weight 1.00

Mrs S has atrial fibrillation, hypertension and hypercholesterolemia. Her current medications include warfarin, bisoprolol and simvastatin. She has been prescribed a 5 day course of antibiotics, amoxicillin and clarithromycin for her community acquired lower respiratory tract infection. Which of the following is correct and most likely to cause a significant problem:

Select one:

- a. No extra monitoring of INR is required whilst Mrs S is on amoxicillin and clarithromycin.
- b. Close monitoring of INR is required as clarithromycin can reduce levels of warfarin.
- c. Amoxicillin and clarithromycin is contraindicated in this patient
- d. Simvastatin does not interact with clarithromycin.
- e. Close monitoring of INR is required as clarithromycin can increase levels of warfarin.

Certainty : C=1 (Unsure: <67%) C=2 (Mid: >67%) C=3 (Quite sure: >80%)

Check

Question **17**

Not complete

Weight 1.00

An elderly gentleman develops AKI during admission: Which of these medications is most likely to have caused renal impairment

Select one:

- a. Vancomycin
- b. Salbutamol
- c. Bisoprolol
- d. Acenocoumarol
- e. Phenytoin

Certainty : C=1 (Unsure: <67%) C=2 (Mid: >67%) C=3 (Quite sure: >80%)

Check

Question **18**

Not complete

Weight 1.00

Mrs E is started on gentamicin and vancomycin for infective endocarditis. She is 5ft 2 and weighs 120kg. Which of the following is **incorrect** regarding gentamicin?

Select one:

- a. Mrs E's actual weight should be used to determine the gentamicin dose.
- b. Mrs E's ideal body weight should be used to determine the gentamicin dose.
- c. Gentamicin dosing is dependent on renal function.
- d. Gentamicin is a narrow therapeutic range medicine and requires monitoring.
- e. Gentamicin can cause irreversible ototoxicity.

Certainty : C=1 (Unsure: <67%) C=2 (Mid: >67%) C=3 (Quite sure: >80%)

Check

Question **19**

Not complete

Weight 1.00

Which of the following side effects of antibiotics means that the same antibiotic should not be given again to that patient in the absence of further workup? **(Select all that apply)**

Select one or more:

- a. Wheezing or breathing difficulties
- b. Rash
- c. Oral or vaginal thrush
- d. Swelling of the mouth or tongue
- e. Loose stools
- f. Nausea/ loss of appetite

Certainty : C=1 (Unsure: <67%) C=2 (Mid: >67%) C=3 (Quite sure: >80%)

Check

Question **20**

Not complete

Weight 1.00

In a patient with ischaemic heart disease on that requires clarithromycin for Community Acquired Pneumonia which of the following medications is most likely to contribute to a significant drug-drug interaction:

Select one:

- a. Atorvastatin
- b. Furosemide
- c. Clopidogrel
- d. Aspirin

Certainty : C=1 (Unsure: <67%) C=2 (Mid: >67%) C=3 (Quite sure: >80%)

Check

Question **21**

Not complete

Weight 1.00

Which of the following are potentially inappropriate antimicrobial combinations due to overlaps in antimicrobial cover? Tick all that apply

Select one or more:

- a. clindamycin and metronidazole, provided there is no *Clostridium difficile* infection
- b. cefalexin and metronidazole
- c. Co-amoxiclav & metronidazole, provided there is no *Clostridium difficile* infection
- d. Co-amoxiclav & flucloxacillin

Certainty : C=1 (Unsure: <67%) C=2 (Mid: >67%) C=3 (Quite sure: >80%)

Check

Question 22
Not complete
Weight 1.00

Which of the following constitute good antimicrobial stewardship principles when reviewed within 24-72 hours:

Select one or more:

- [ ] a. Antibiotics stopped when initially started for possible lower respiratory tract infection but chest X-ray clear, CRP 32mg/L, WCC 8.2, afebrile and improved respiratory function
- [ ] b. Antibiotic treatment is escalated to Meropenem in a patient with an E.coli bacteriaemia not responding to empirical treatment for sepsis and with a history of ESBL infection with a plan to review again in 72 hours
- [ ] c. Patient switched from IV benzylpenicillin to oral amoxicillin in a patient clinically responding to treatment for a probable community acquired pneumonia for a total of 7 days.
- [ ] d. Patient referred to Outpatient Antimicrobial Management Team (OPAT) for continuation of treatment for cellulitis in a clinically stable patient aged 42 years of age with a plan to switch antibiotics as per the local OPAT guidelines with a tentative course duration and review date.

Certainty : ○ C=1 (Unsure: <67%) ○ C=2 (Mid: >67%) ○ C=3 (Quite sure: >80%)

Check

Question 23
Not complete
Weight 1.00

An 48-72 hour review is a key part of the Start smart and focus initiative which of the following is **not** correct

Select one:

○ a. If the patient is still having temperatures a change in antibiotics may not be needed if there is a deep seated source of infection

○ b. Orals are not suitable if the CRP is above 150

○ c. Antibiotics should be stopped even if a course has not been completed if infection can be ruled out

Certainty : ○ C=1 (Unsure: <67%) ○ C=2 (Mid: >67%) ○ C=3 (Quite sure: >80%)

Check

Question 24
Not complete
Weight 1.00

What are the fundamental questions in Quality Improvement?

Select one or more:

- [ ] a. What changes can we make that will result in improvement?
- [ ] b. What are we trying to accomplish?
- [ ] c. How will we know that a change is an improvement?

Certainty : ○ C=1 (Unsure: <67%) ○ C=2 (Mid: >67%) ○ C=3 (Quite sure: >80%)

Check

Question 25
Not complete
Weight 1.00

The following tools and techniques can all be used for Quality Improvement. Which is the preferred tool/technique in healthcare?

Select one:

○ a. Variation

○ b. Process mapping

○ c. PDSA cycle

○ d. Demand and capacity

Certainty : ○ C=1 (Unsure: <67%) ○ C=2 (Mid: >67%) ○ C=3 (Quite sure: >80%)

Check

Question 26
Not complete
Weight 1.00

With whom does the responsibility for improvement ultimately lie?

Select one:

○ a. Only doctors, nurses or pharmacists

○ b. All staff

○ c. Dedicated improvement officer

○ d. Senior management

Certainty : ○ C=1 (Unsure: <67%) ○ C=2 (Mid: >67%) ○ C=3 (Quite sure: >80%)

Check

Question 27
Not complete
Weight 1.00

What questions do you think you should ask yourself before you start an improvement project?

Select one or more:

- ☐ a. Do other people carry out the same activity in the same way?
- ☐ b. What do you compare your effectiveness against?
- ☐ c. What do you already know about it?
- ☐ d. How will you measure your effectiveness in that activity?

Certainty : ○ C=1 (Unsure: <67%) ○ C=2 (Mid: >67%) ○ C=3 (Quite sure: >80%)

Check

Question 28
Not complete
Weight 1.00

Pick the top 3 determinants for behaviour.

Select one or more:

- ☐ a. Knowledge
- ☐ b. I don't know
- ☐ c. Skills
- ☐ d. Capability
- ☐ e. Opportunity
- ☐ f. Motivation

Certainty : ○ C=1 (Unsure: <67%) ○ C=2 (Mid: >67%) ○ C=3 (Quite sure: >80%)

Check

Question 29
Not complete
Weight 1.00

Match the following examples with the associated determinant of behaviour.

A nurse washing her hands consistently every since her training on the five moments of hand hygiene decades ago	Choose...
A clinician thinking about the information they saw in a leaflet about the pros and cons of using a certain drug over another the next time that they treat a patient.	Choose...
A doctor is struggling to incorporate good practice that they learnt at a training course because his colleagues don't seethe point in doing it that way.	Choose...

Certainty : ○ C=1 (Unsure: <67%) ○ C=2 (Mid: >67%) ○ C=3 (Quite sure: >80%)

Check

Question 30
Not complete
Weight 1.00

Which of the following are true?

Select one or more:

- ☐ a. Conducting an audit may done as a part of Quality Improvement, but is not Quality Improvement in itself.
- ☐ b. Once you have implemented an intervention, you have conducted Quality Improvement
- ☐ c. Quality Improvement and audit is the same thing
- ☐ d. Measurement for Quality Improvement may involve smaller scale data collection done more frequently (e.g. weekly) that allow you to test the impact of change in real time as opposed to collecting one or two large retrospective samples of data in isolation.

Certainty : ○ C=1 (Unsure: <67%) ○ C=2 (Mid: >67%) ○ C=3 (Quite sure: >80%)

Check

◄ Test of knowledge quiz: pre-training (hidden)

Jump to...

Knowledge and Capability Guide ►
