# Supplementary material for: Outcomes of a National, Cross-Sector Antimicrobial Stewardship Training Initiative for Pharmacists in England
Source: Pharmacy (Basel). 2021 Oct 10;9(4):165. doi: 10.3390/pharmacy9040165 (PMC8544733; doi:10.3390/pharmacy9040165)
Supplement: Supplementary file 1 [file pharmacy-09-00165-s001.zip › Supplementary materials (S4) - Knowledge quiz (cohorts 2 and 3).pdf]

Question **1**

Not yet answered

Marked out of 1.00

Which of the following statements are **TRUE (Select all that apply**):

Select one or more:

- [ ] a. Antibiotics are effective against viruses.
- [ ] b. Antibiotics are effective against cold and flu.
- [ ] c. Unnecessary use of antibiotics [can] make them become ineffective.
- [ ] d. Taking antibiotics has associated side effects or risks such as diarrhoea, colitis, allergies.
- [ ] e. Every person treated with antibiotics is at an increased risk of antibiotic resistant infection.
- [ ] f. Antibiotic resistant bacteria can spread from person to person.
- [ ] g. Healthy people can carry antibiotic resistant bacteria.
- [ ] h. The use of antibiotics to stimulate growth in farm animals is legal in the EU.

Question **2**

Not yet answered

Marked out of 1.00

Which of the following are examples of collaborative AMS roles and responsibilities that are shared by all pharmacy, medical and nursing staff? **(Select all that apply)**

Select one or more:

- [ ] a. Education and involvement of patients
- [ ] b. Administration of fluids and antimicrobial therapy, wound care
- [ ] c. Adherence to Infection Prevention and Control practice
- [ ] d. Timely initiation or administration of antimicrobial therapy
- [ ] e. Document indication, dose and duration in patient record
- [ ] f. Establish diagnosis and management plan for patient with suspected infection
- [ ] g. Monitoring and review of therapy in relation to allergy status, suitability for IV to oral switch

Question **3**

Not yet answered

Marked out of 1.00

Antimicrobial Stewardship is defined as:

Select one:

- ○ a. Stewardship of antimicrobial prescribing practices through prescriber self-regulation and an opt-in approach to AMS programmes and standards
- ○ b. A systems-wide approach to promoting the judicious use of antibiotics, but not antifungals or antivirals
- ○ c. A holistic approach to reducing antimicrobial consumption to minimise the cost burden of high volume use.
- ○ d. Organisational or healthcare-system-wide approach to promoting and monitoring judicious use of antimicrobials to preserve their future effectiveness

Question **4**

Not yet answered

Marked out of 1.00

The PHE/AHRAI have outlined Antimicrobial Stewardship competency standards. Which of the following are in this list? [Select 7]

Select one or more:

- [ ] a. Increase duration and broaden antibiotic therapy if there is inadequate response within 72 hours.
- [ ] b. Do not alter the choice of antibiotic for the remainder of the course if there has been a good response to it.
- [ ] c. Use only single doses of antimicrobials for surgical or other prophylaxis where it has been shown to be effective, unless the procedure has been prolonged, there has been excessive blood loss, or published national recommendations suggest otherwise.
- [ ] d. In primary care: use the TARGET antibiotics toolkit and HPA national guidance; In secondary care: follow the Start Smart-then Focus guidance
- [ ] e. Educating patients and their carers, nurses and other supporting staff as to when antibiotics are not required, and complying with the duration and frequency of administration of their prescribed antimicrobial.
- [ ] f. Avoid unnecessary use of broad-spectrum antimicrobials
- [ ] g. Document the clinical indication, route, dose, duration and review date of antimicrobials in the patient record and/or prescription
- [ ] h. Switch to the correct antimicrobial when susceptibility testing indicates resistance or to a more cost-effective antimicrobial that is also compatible with the clinical presentation
- [ ] i. Use local guidelines to initiate prompt effective antimicrobial treatment in patients with life-threatening infections

Question **5**

Not yet answered

Marked out of 1.00

Which of the following activities are aligned with the UK AMR Action plan priorities for optimising use of antimicrobials in humans? **(Select all the apply)**

Select one or more:

- a. Using electronic prescribing data and systems to provide feedback and guidance on prescribing.
- b. Enhanced review of long-term and repeat antimicrobial prescriptions.
- c. Addressing look-alike-sound-alike medication errors relating to antimicrobials.
- d. Optimising generic brand switches for antimicrobials to generate cost savings.
- e. Raising public awareness to encourage self-care and reduce expectations of antibiotics.
- f. Development of NHS trust strategy to set a 2 year reduction target for 'Reserve' and 'Watch' antimicrobial usage.
- g. Enhanced pharmacy review of dose and duration of antimicrobial prescriptions.

Question **6**

Not yet answered

Marked out of 1.00

The UK has set a target of a 10% reduction in the use of 'reserve' and 'watch' antimicrobials in hospitals. Why should all practitioners be especially vigilant when using these medicines?
**(Select all that apply)**

Select one or more:

- a. These medicines should only be prescribed for specific indications, in some instances for severe circumstances, or when other options have failed.
- b. These medicines are harder to access with only small reserves available in the health system.
- c. Reducing unnecessary or inappropriate use of these medicines will reduce the burden of resistance at a population level.
- d. Using these medicines in patients increases their risk of being colonised or infected with multi-resistant organisms and C. difficile compared with lower risk antimicrobials.
- e. These are expensive medicines that reduce the ability to fund other aspects of healthcare delivery.

Question **7**

Not yet answered

Marked out of 1.00

Which of the following statements about healthcare-associated infections are correct?
**(Select all that apply)**

Select one or more:

- a. Surgical prophylaxis requires a balanced approach as antimicrobial prophylaxis can prevent surgical site infections if used optimally but cause adverse effects if used inappropriately.
- b. Hospital acquired pneumonia can be prevented by getting patients sat out of bed, or upright
- c. Reducing C. difficile acquisition is less relevant for AMS programmes as this is primarily driven by Infection Control.
- d. Healthcare-associated infections are centred around hospitals and therefore do not present in the primary care setting

Question **8**

Not yet answered

Marked out of 1.00

When should you dip a urinary catheter?

Select one:

- a. When you have no other source of reliable urine.
- b. Never
- c. When you have an unclear diagnosis and suspect UTI.

Question **9**

Not yet answered

Marked out of 1.00

In a patient with ischaemic heart disease that requires clarithromycin for Community Acquired Pneumonia which of the following medications is most likely to contribute to a significant drug-drug interaction:

Select one:

- a. Aspirin
- b. Clopidogrel
- c. Furosemide
- d. High dose atorvastatin

Question **10**

Not yet answered

Marked out of 1.00

Why are urinary catheters problematic for UTI? **(Select all that apply)**

Select one or more:

- a. A biofilm can be created which harbours microbes antibiotics cannot reach.
- b. Increases the risk of pathogenic bacteria being introduced into the body and the risk of urinary tract infection, including urosepsis.
- c. Requires frequent antibiotic prophylaxis for catheter changes to prevent infection.
- d. Stagnant, cloudy urine in situ requiring frequent routine microbiological testing to rule-out infection.

Question **11**
Not yet answered
Marked out of 1.00

Which of the following are correct in relation to the management of diabetic foot infection and cellulitis/erisipelas in accordance to NICE guidelines? **(Select all that apply):**

Select one or more:

- a. Flucloxacillin is the preferred first line treatment, followed by clarithromycin, erythromycin or doxycycline for both cellulitis/erisipelas and mild diabetic foot infection.
- b. Considerations for referral to hospital include ulceration with ischaemia, and infection near the eyes or nose.
- c. A positive wound swab result should always be treated.
- d. The recommended duration of antibiotic therapy for mild disease is 5-7 days.
- e. A diabetic foot ulcer with swelling and no other symptoms is classified as a diabetic foot infection.
- f. The severity of a diabetic foot infection is determined by how long the ulcer has been present for.

Question **12**
Not yet answered
Marked out of 1.00

Choose the most appropriate course of action. You see a patient with blocked urinary catheter, urine cultures have grown > 10^5 CFU/mL Pseudomonas species.

Select one:

- a. IV ceftazidime
- b. Oral ciprofloxacin
- c. Co-amoxiclav if there are signs of infection.
- d. No antibiotic therapy unless there are signs of systemic infection or sepsis

Question **13**
Not yet answered
Marked out of 1.00

Which of the following are in line with the NICE antimicrobial guidelines for acute cough? **(Select all that apply)**

Select one or more:

- a. A back-up prescription for clarithromycin 500mg twice a day for a 55 year old patient who is also taking oral corticosteroids, with instructions to fill it if symptoms worsen rapidly or significantly
- b. Amoxicillin 500mg three times a day for 5 days for a 30 year old male who is systemically well and has no other medical conditions
- c. Levofloxacin 500mg daily for 10 days in an asthmatic patient.
- d. Doxycycline 200mg on the first day, followed by 100mg daily for a further 4 days for a 80 year old diabetic patient

Question **14**
Not yet answered
Marked out of 1.00

Match the following respiratory condition with the corresponding statement:

| | |
|---|---|
| Infection of the main airways of the lungs causing them to become irritated and inflamed. The main symptom is a hacking cough which may bring up phlegm. | Choose... |
| Sustained worsening of symptoms from a stable state in a patient with chronic lung disease which can be caused by a range of factors including infection. Clinical indicators exist to determine the likelihood of bacterial involvement that would benefit from antibiotics. | Choose... |
| Infection leading to the alveolar tissue in one or both of the lungs to be inflamed and fill up with fluid/pus. Chest x-rays are used in hospital to confirm diagnosis whilst in primary care diagnosis is mostly based on clinical assessment of symptoms (e.g. fever, chest pain, chest examination, shortness of breath, decreased oxygen saturation). | Choose... |
| Most commonly caused by Streptococus pneumoniae, although other organisms can be implicated as well. Severity can be assessed using the CRB65 or CURB65 score depending on the healthcare setting. | Choose... |
| Pneumonia that develops 48 hours or more after hospital admission. May involve bacteria that usually reside in the gastro-intestinal system as well as the respiratory tract | Choose... |

Question **15**
Not yet answered
Marked out of 1.00

Asymptomatic bacteriuria should always be treated in:

Select one:

- a. Elderly
- b. Pregnant women
- c. Children
- d. All of the above

Question 16
Not yet answered
Marked out of 1.00

Which of the following are risk factors for Clostridium difficile infection or colonisation? **(Select all that apply)**

Select one or more:

- a. Proton pump inhibitors.
- b. Previous Clostridium difficile infection.
- c. Elderly patient.
- d. Broad spectrum and high-risk antibiotics.
- e. Delayed antimicrobial administration/initiation.
- f. Immunocompromise.

Question 17
Not yet answered
Marked out of 1.00

The number of gram negative (such as E. coli) blood stream infections in the UK is increasing. Which of the following interventions may be suitable ways to tackle this?

**(Select all that apply)**

Select one or more:

- a. Using broader spectrum antibiotics across the board to minimise antibiotic mismatch as much as possible.
- b. Using long term antibiotic prophylaxis for all care home residents with 2 or more UTIs in the last 12 months.
- c. Timely removal of urinary catheters.
- d. Timely drainage or surgical management of deep seated infections, where possible and appropriate.
- e. Asking patients with abdominal and urinary tract infections about recent international travel and previous infections to assess the risk of multi-resistant organism involvement.

Question 18
Not yet answered
Marked out of 1.00

Which age group is more likely to develop E. coli bacteraemia?

Select one:

- a. <65 years
- b. >80 years
- c. 65-80 years

Question 19
Not yet answered
Marked out of 1.00

Which of the following statements are correct in relation to infection markers:
**(Select all that apply)**

Select one or more:

- a. A raised WCC and neutrophils may indicate increased immune system activity due to infection.
- b. Deranged LFTs, impaired glycaemic control, confusion, hypotension, decreased platelets and pain are examples of how infection can manifest as inflammation and organ dysfunction.
- c. WCC and neutrophils will always be raised when there is an infection.
- d. Heavy growth of bacteria in a microbiological culture will always mean that there is an infection.
- e. Infection markers need to be interpreted in the context of a patient's symptoms and clinical history as they do not always indicate infection.

Question 20
Not yet answered
Marked out of 1.00

Which of the following are examples of antimicrobials in the 'Watch' AwARE category?
**(Select all that apply)**

Select one or more:

- a. Cefalexin
- b. Azithromycin
- c. Fosfomycin IV
- d. Nitrofurantoin
- e. Co-amoxiclav
- f. Fosfomycin oral

Question 21
Not yet answered
Marked out of 1.00

What is the most appropriate source of information to use for antibiotic choice, dose and duration?

Select one:

- a. NICE antimicrobial prescribing guidelines should override local guidelines in all instances.
- b. Local prescribing preferences that have been agreed upon and written down should be followed.
- c. Peer reviewed, local guidelines that incorporate local data and are endorsed by your organisation should be used, or if the abscence of these, the NICE antimicrobial prescribing guidelines.
- d. BNF and SPCs should be the first port of call.

Question **22**
Not yet answered
Marked out of 1.00

Match the most appropriate course of action to the following penicillin allergy/ADR histories:

- Non-severe reaction, such as mild maculopauplar or benign childhood rash. — Choose...
- Severe cutaneous reactions (e.g. SJS, TEN, extensive hives), DRESS, anaphylaxis, interstitial nephritis, severe liver injury. — Choose...
- Nausea, vomiting, headache, dizziness, diarrhoea — Choose...

Question **23**
Not yet answered
Marked out of 1.00

Match the following antibiotics or antibiotic combinations to the corresponding statement about their microbial coverage:

| | |
|---|---|
| Linezolid, cotrimoxazole, clindamycin, doxycycline | Choose... |
| Co-amoxiclav | Choose... |
| Flucloxacillin, dicloxacillin | Choose... |
| Doxycycline, clarithromycin, macrolides | Choose... |
| Metronidazole | Choose... |
| Cefalexin | Choose... |
| Amoxicillin, doxycycline | Choose... |

Question **24**
Not yet answered
Marked out of 1.00

A pharmacist conducts regular antibiotic prescribing audits and discusses the results with the prescribers to evaluate areas for improvement. Is this an example of Data-driven care/Data for action, and why?

Select one:

- a. Yes - because it involves the use of data within practice to improve patient outcomes and safety.
- b. No - because Data for Action specifically applies to quantitative and nominal data.
- c. No - because there hasn't been any input from an informatics specialist.
- d. Yes - because the data is being reported and shared.
- e. Yes - because patient-specific prescribing data is being collected.
- f. No - because Data-driven care only applies to the use of big data.

Question **25**
Not yet answered
Marked out of 1.00

Which of the following tools mainly uses primary care reimbursement data to illustrate prescribing? **(Select all that apply)**

Select one or more:

- a. NHS Model Hospital
- b. PrescQIPP Data Hub
- c. PHE Fingertips
- d. ePACT2
- e. OpenPrescribing.net
- f. Rx-Info Define
- g. PrescQIPP Antimicrobial Stewardship Hub

**Question 26**
Not yet answered
Marked out of 1.00

Which of the following tools are openly accessible? **(Select all that apply)**

Select one or more:

- a. PrescQIPP Antimicrobial Stewardship Hub
- b. Rx-Info Define
- c. PHE Fingertips
- d. PrescQIPP Data Hub
- e. NHS Model Hospital
- f. ePACT2
- g. OpenPrescribing.net

**Question 27**
Not yet answered
Marked out of 1.00

What are the 3 fundamental questions in Quality Improvement?
**(Select all that apply)**

Select one or more:

- a. What changes can we make that will result in improvement?
- b. How do we compare with others?
- c. How will we know that a change is an improvement?
- d. What are we trying to accomplish?
- e. How can we keep things the way they are?

**Question 28**
Not yet answered
Marked out of 1.00

The following tools and techniques can all be used for Quality Improvement. Match the following methodologies with their corresponding statements:

| | |
|---|---|
| - Tools to help provide a more objective understanding of the causes of a problem to avoid before making changes based on assumptions. | Choose... |
| - Framework for implementing intervention(s) to tackle a clearly defined problem with specific aims, objectives and measures. It ensures that interventions are adequately tested and evaluated to provide iterative and cyclical learning that informs ongoing implementation. | Choose... |
| - An approach to evaluate performance against agreed upon standards and analyse why deviation from the standard occurs | Choose... |
| - Tools to analyse workflow activity to evaluate delays within a system and devise strategies to improve efficiency. | Choose... |

**Question 29**
Not yet answered
Marked out of 1.00

With whom does the responsibility for improvement ultimately lie?

Select one:

- a. Dedicated improvement officer
- b. Only doctors, pharmacists and nurses
- c. Senior management
- d. All staff

**Question 30**
Not yet answered
Marked out of 1.00

What questions do you think you should ask yourself before you start an improvement project?
**(Select all that apply)**

Select one or more:

- a. How can I minimise awareness of this project so that the scope can be kept manageable?
- b. Do other people carry out the same activity in the same way?
- c. How will I measure effectiveness in that activity?
- d. What do I compare effectiveness against?
- e. What do I already know about it?
- f. How can this streamline my workload?

Question **31**
Not yet answered
Marked out of 1.00

Choose the correct descriptors for the 3 COM-B domains that determine behaviour?
**(Select all that apply)**

Select one or more:

- [ ] a. Capability - psychological and physical knowledge and skills.
- [ ] b. Motivation - Beliefs about consequences, emotional and habitual responses.
- [ ] c. Capability - Ability to understand and analyse the intentions of others in relation to their behaviour.
- [ ] d. Knowledge and capability - cognitive and analytical skills, awareness of ideas and concepts.
- [ ] e. Opportunity - influence of the social (e.g. support) and physical (e.g. access, resource, time) environment.
- [ ] f. Motivation - confidence and drive to attain goals and improve outcomes.

Question **32**
Not yet answered
Marked out of 1.00

Match the following examples with the associated determinant of behaviour.

| | |
|---|---|
| - A nurse washing her hands consistently ever since their training on the five moments of hand hygiene decades ago. | Choose... |
| - A clinician thinking about the information they saw in a leaflet about the pros and cons of using a certain drug over another the next time that they treat a patient. | Choose... |
| - A doctor is struggling to incorporate good practice that they learnt at a training course because their colleagues don't see the point in doing it that way. | Choose... |

Question **33**
Not yet answered
Marked out of 1.00

Which of the following are true? **(Select all that apply)**

Select one or more:

- [ ] a. Quality Improvement and audit is the same thing
- [ ] b. Measurement for Quality Improvement using PDSA methodology may involve smaller scale data collection done more frequently (e.g. weekly) that allow you to test the impact of change in real time as opposed to collecting one or two large retrospective samples of data in isolation.
- [ ] c. Conducting an audit may be done as a part of Quality Improvement, but is not Quality Improvement in itself.
- [ ] d. Once you have implemented an intervention, you have conducted Quality Improvement

◄ Guidance and instructions for education/training session

Jump to...

Self-assessment questionnaire ►
