# Supplementary material for: Outcomes of a National, Cross-Sector Antimicrobial Stewardship Training Initiative for Pharmacists in England
Source: Pharmacy (Basel). 2021 Oct 10;9(4):165. doi: 10.3390/pharmacy9040165 (PMC8544733; doi:10.3390/pharmacy9040165)
Supplement: Supplementary file 1 [file pharmacy-09-00165-s001.zip › Supplementary materials (S5) - Learner improvement project activities.pdf]

Learner improvement project activities (n=83)

Sector	Topic category	Improvement topic	Intervention or change idea	Intervention category	COM-B	Secured input or support from colleagues to undertake QI project	Collected data that improved understanding of problem	Gained new learning from implementing and evaluating a test of change (PDSA cycle)	Shared information and awareness about initiative outside of core team	Educated others to develop knowledge skills, attitudes
Community Health Trust	UTI	UTI prescribing	Education and promotion	Education and promotion	C	Yes	Yes	Yes	Yes	Yes
Primary Care	Broad spectrum antimicrobials	Reducing prescribing of broad-spectrum antibiotics in GP surgeries	Audit and feedback facilitated supported through EMIS	Prospective audit and feedback	Multiple	Yes		Yes		Yes
Primary Care	Lower respiratory tract infections	Prescribing variation in primary care: Utilising resources to reduce inappropriate prescribing with a focus on respiratory tract infection	TARGET 'treat your infection' leaflet F12 protocol on EMIS	Use of practice aids	O	Yes		Yes	Yes	
Primary Care	Lower respiratory tract infections	Respiratory tract infection antibiotic prescribing	Access to TARGET leaflets in prescribing software	Use of practice aids	O	Yes		Yes	Yes	Yes

Learner improvement project activities (n=83)

Sector	Topic category	Improvement topic	Intervention or change idea	Intervention category	COM-B	Secured input or support from colleagues to undertake QI project	Collected data that improved understanding of problem	Gained new learning from implementing and evaluating a test of change (PDSA cycle)	Shared information and awareness about initiative outside of core team	Educated others to develop knowledge skills, attitudes
Primary Care	Broad spectrum antimicrobials	High-risk antimicrobial audit and feedback in primary care	Audit and feedback (using validated tool from elsewhere	Prospective audit and feedback	Multiple	Yes		Yes	Yes	
Primary Care	Lower respiratory tract infections	Reducing inappropriate prescribing of antibiotic rescue packs for Chronic Obstructive Pulmonary Disease	New procedures for management and review of COPD antibiotic rescue pack prescriptions	Restriction	O	Yes		Yes	Yes	Yes
Primary Care	Broad spectrum antimicrobials	Broad spectrum antibiotic prescribing	*C. Diff* review form	Prospective audit and feedback	Multiple	Yes	Yes			
Secondary Care	Documentation of indication	Documentation of antibiotic indication	Education sessions; future plans for EPMA alert	Electronic prescribing	Multiple	Yes		Yes	Yes	Yes

Learner improvement project activities (n=83)

Sector	Topic category	Improvement topic	Intervention or change idea	Intervention category	COM-B	Secured input or support from colleagues to undertake QI project	Collected data that improved understanding of problem	Gained new learning from implementing and evaluating a test of change (PDSA cycle)	Shared information and awareness about initiative outside of core team	Educated others to develop knowledge skills, attitudes
Secondary Care	Diagnostic stewardship	Reducing the number of urine dips requested in patients over 65 years to diagnose urinary tract infections in acute medicine	Education and promotion, inviting staff to sign a pledge	Role modelling	O	Yes	Yes	Yes	Yes	Yes
Secondary Care	UTI	Improving prescribing practices in patients with UTI	Education and promotion	Education and promotion	C	Yes	Yes	Yes	Yes	Yes
Secondary Care	48-72 hour review	48-72 hour antibiotic reviews	Audit and feedback facilitated by mobile app	Prospective audit and feedback	Multiple	Yes		Yes	Yes	
Secondary Care	48-72 hour review	72 hour antibiotic reviews	Audit and feedback facilitated by mobile app	Prospective audit and feedback	Multiple	Yes		Yes	Yes	

Learner improvement project activities (n=83)

Sector	Topic category	Improvement topic	Intervention or change idea	Intervention category	COM-B	Secured input or support from colleagues to undertake QI project	Collected data that improved understanding of problem	Gained new learning from implementing and evaluating a test of change (PDSA cycle)	Shared information and awareness about initiative outside of core team	Educated others to develop knowledge skills, attitudes
Secondary Care	Lower respiratory tract infections	Clarithromycin prescribing in mild-moderate community acquired pneumonia	Use of CURB score documentation as a prompt	Prompts and reminders	M	Yes	Yes	Yes		
Secondary Care	Lower respiratory tract infections	Community acquired pneumonia	Intervention package: CURB score documentation , ward stock changes	Restriction	O	Yes	Yes	Yes		
Secondary Care	Lower respiratory tract infections	Co-amoxiclav prescribing in community acquired pneumonia	Prompt documentation of CURB score and review plan	Prompts and reminders	M	Yes	Yes	Yes	Yes	

Learner improvement project activities (n=83)

Sector	Topic category	Improvement topic	Intervention or change idea	Intervention category	COM-B	Secured input or support from colleagues to undertake QI project	Collected data that improved understanding of problem	Gained new learning from implementing and evaluating a test of change (PDSA cycle)	Shared information and awareness about initiative outside of core team	Educated others to develop knowledge skills, attitudes
Secondary Care	Timely antibiotic administration	Reducing the time from prescribing to administration of restricted antimicrobials in the inpatient setting	New procedure for ordering and dispensing of restricted antimicrobials	Guideline or procedure	O	Yes	Yes	Yes	Yes	Yes
Secondary Care	Timely antibiotic administration	Time to administration of IV antibiotics for diabetes foot infections in the Diabetes and Endocrine Unit	New procedure for initiating antibiotic therapy in the unit	Guideline or procedure	O	Yes	Yes	Yes	Yes	

Learner improvement project activities (n=83)

Sector	Topic category	Improvement topic	Intervention or change idea	Intervention category	COM-B	Secured input or support from colleagues to undertake QI project	Collected data that improved understanding of problem	Gained new learning from implementing and evaluating a test of change (PDSA cycle)	Shared information and awareness about initiative outside of core team	Educated others to develop knowledge skills, attitudes
Secondary Care	48-72 hour review	The impact of a new antimicrobial drug chart on prescribed antibiotics at 48-72 hours	New antimicrobial prescribing chart	Use of practice aids	O	Yes		Yes	Yes	Yes
Secondary Care	Therapeutic Drug Monitoring	Triazole therapeutic drug monitoring	Future plans to introduce SOP with prospective audit and feedback	Guideline or procedure	C	Yes	Yes		Yes	Yes

Learner improvement project activities (n=83)

Sector	Topic category	Improvement topic	Intervention or change idea	Intervention category	COM-B	Secured input or support from colleagues to undertake QI project	Collected data that improved understanding of problem	Gained new learning from implementing and evaluating a test of change (PDSA cycle)	Shared information and awareness about initiative outside of core team	Educated others to develop knowledge skills, attitudes
Community pharmacy	Patient understanding of antimicrobials	Community pharmacy antibiotic dispensing (To improve patient understanding and self-management of conditions requiring antibiotics)	Community Pharmacy Antibiotic checklist	Use of practice aids	C		Yes			
Community pharmacy	Upper respiratory tract infections	Self-limiting URTIs (TBA)	Academic detailing/education	Education and promotion	C					
Community pharmacy	Upper respiratory tract infections	Self-limiting URTIs (TBA)	Academic detailing/education	Education and promotion	C	Yes				

Learner improvement project activities (n=83)

Sector	Topic category	Improvement topic	Intervention or change idea	Intervention category	COM-B	Secured input or support from colleagues to undertake QI project	Collected data that improved understanding of problem	Gained new learning from implementing and evaluating a test of change (PDSA cycle)	Shared information and awareness about initiative outside of core team	Educated others to develop knowledge skills, attitudes
Community pharmacy	Antibiotic duration	Duration of antibiotic prescriptions (Improve GP prescribing of antibiotics in line with NICE guidance)								
Community pharmacy	Broad spectrum antimicrobials	Co-amoxiclav prescribing (To reduce inappropriate prescribing)	Prospective audit and feedback	Prospective audit and feedback	Multiple	Yes			Yes	
Community pharmacy	Upper respiratory tract infections	Self-limiting URTIs (Reduce patient demand for GP practices for antibiotics)	Community Pharmacy Antibiotic checklist	Use of practice aids	C					Yes

Learner improvement project activities (n=83)

Sector	Topic category	Improvement topic	Intervention or change idea	Intervention category	COM-B	Secured input or support from colleagues to undertake QI project	Collected data that improved understanding of problem	Gained new learning from implementing and evaluating a test of change (PDSA cycle)	Shared information and awareness about initiative outside of core team	Educated others to develop knowledge skills, attitudes
Primary care	UTI	UTI (Improve appropriateness of UTI prescribing)								
Primary care	UTI	UTI (Reduce frequency of UTI diagnosis associated with urine dipstick testing)	Sharing and benchmarking practice-level data	Social norms benchmarking	O	Yes				
Primary care	Broad spectrum antimicrobials	Broad spectrum antibiotics (Improve appropriateness of broad-spectrum antibiotic prescribing)								

Learner improvement project activities (n=83)

Sector	Topic category	Improvement topic	Intervention or change idea	Intervention category	COM-B	Secured input or support from colleagues to undertake QI project	Collected data that improved understanding of problem	Gained new learning from implementing and evaluating a test of change (PDSA cycle)	Shared information and awareness about initiative outside of core team	Educated others to develop knowledge skills, attitudes
Primary care	UTI	UTI (To improve appropriateness of UTI prescribing in the elderly (>70 years))								
Primary care	Other	Antibiotic appropriateness (To improve protocol prescribing on electronic system)	Academic detailing/education	Education and promotion	C					
Primary care	Lower respiratory tract infections	Clarithromycin (To reduce inappropriateness of clarithromycin prescribing)								

Learner improvement project activities (n=83)

Sector	Topic category	Improvement topic	Intervention or change idea	Intervention category	COM-B	Secured input or support from colleagues to undertake QI project	Collected data that improved understanding of problem	Gained new learning from implementing and evaluating a test of change (PDSA cycle)	Shared information and awareness about initiative outside of core team	Educated others to develop knowledge skills, attitudes
Primary care	UTI	UTI (Reduce overdiagnosis of UTI in care homes due to dipstick testing)	Adapt existing UTI assessment form	Use of practice aids	C	Yes			Yes	
Primary care	UTI	UTI (To improve prescribing guideline compliance)	Academic detailing/education	Education and promotion	C	Yes			Yes	
Primary care	UTI	UTI (Reduce trimethoprim/nitrofurantoin prescribing rate)	EMIS prompt	Prompts and reminders	M	Yes				

Learner improvement project activities (n=83)

Sector	Topic category	Improvement topic	Intervention or change idea	Intervention category	COM-B	Secured input or support from colleagues to undertake QI project	Collected data that improved understanding of problem	Gained new learning from implementing and evaluating a test of change (PDSA cycle)	Shared information and awareness about initiative outside of core team	Educated others to develop knowledge skills, attitudes
Primary care	Upper respiratory tract infections	Sore throat (Improve formulary compliance and use of risk stratification tool (FEVERPAIN))	Incorporate FEVERPAIN tool into EMIS	Use of practice aids	C	Yes				
Primary care	Lower respiratory tract infections	COPD (Reduce inappropriate prescribing of antibiotic rescue packs)	Implement/change guidance	Guideline or procedure	O	Yes				
Primary care	Antibiotic allergy	Antibiotic allergy (Increase proportion of penicillin allergic patients with this documented in MR)	Train healthcare assistants to obtain information on antibiotic allergies	Education and promotion	C					

Learner improvement project activities (n=83)

Sector	Topic category	Improvement topic	Intervention or change idea	Intervention category	COM-B	Secured input or support from colleagues to undertake QI project	Collected data that improved understanding of problem	Gained new learning from implementing and evaluating a test of change (PDSA cycle)	Shared information and awareness about initiative outside of core team	Educated others to develop knowledge skills, attitudes
Primary care	Broad spectrum antimicrobials	Broad spectrum antibiotics (Improve appropriateness of prescribing)	Reminder/prompts	Prompts and reminders	M	Yes				
Primary care	Other	Antibiotic repeat prescribing (Improve regular review of long-term prescriptions)	Reminder/prompts; case-finding	Prompts and reminders	M	Yes				
Primary care	UTI	UTI (Reduce trimethoprim/nitrofurantoin prescribing rate)								
Secondary Care	Broad spectrum antimicrobials	Co-amoxiclav prescribing (Improve guideline compliance)				Yes			Yes	

Learner improvement project activities (n=83)

Sector	Topic category	Improvement topic	Intervention or change idea	Intervention category	COM-B	Secured input or support from colleagues to undertake QI project	Collected data that improved understanding of problem	Gained new learning from implementing and evaluating a test of change (PDSA cycle)	Shared information and awareness about initiative outside of core team	Educated others to develop knowledge skills, attitudes
Secondary Care	Antibiotic allergy	Antibiotic allergy (To reduce incidents due to prescribing of antibiotics where an allergy is documented)	Academic detailing/education	Education and promotion	C					
Secondary Care	Antibiotic duration	Antibiotic duration (Improve documentation of antibiotic duration)	Academic detailing/education	Education and promotion	C	Yes	Yes			
Secondary Care	Lower respiratory tract infections	CAP (To improve frequency of CURB65 score documentation)				Yes			Yes	

Learner improvement project activities (n=83)

Sector	Topic category	Improvement topic	Intervention or change idea	Intervention category	COM-B	Secured input or support from colleagues to undertake QI project	Collected data that improved understanding of problem	Gained new learning from implementing and evaluating a test of change (PDSA cycle)	Shared information and awareness about initiative outside of core team	Educated others to develop knowledge skills, attitudes
Secondary Care	UTI	UTI (Improve appropriateness of UTI prescribing (CQUIN))	Academic detailing/education; prospective audit and feedback, education	Prospective audit and feedback	Multiple	Yes			Yes	
Secondary Care	Therapeutic Drug Monitoring	Antifungal TDM (Increase proportion of TDM in patients who require it)	Implement/change guidance; implement/change procedure(s)	Guideline or procedure	O	Yes			Yes	
Secondary Care	UTI	UTI (Improve review of microbiology samples to guide treatment review)	Academic detailing/education; implement/change procedure(s)	Guideline or procedure	Multiple	Yes			Yes	
Secondary Care	Lower respiratory tract infections	Bronchiectasis (Rationalise duration of therapy)	Implement/change guidance	Guideline or procedure	O	Yes			Yes	

Learner improvement project activities (n=83)

Sector	Topic category	Improvement topic	Intervention or change idea	Intervention category	COM-B	Secured input or support from colleagues to undertake QI project	Collected data that improved understanding of problem	Gained new learning from implementing and evaluating a test of change (PDSA cycle)	Shared information and awareness about initiative outside of core team	Educated others to develop knowledge skills, attitudes
Secondary Care	Other	Gonorrhoea (Improve appropriateness of prescribing)	Implement /change guidance	Guideline or procedure	O					
Secondary Care	Antibiotic wastage	Reducing antibiotic wastage								
Secondary Care	Antibiotic dosing	Gentamicin (To reduce gentamicin-related incidents by ensuring correct prescribing and monitoring)	EPMA alert/prompt	Prompts and reminders	M					
Secondary Care	Broad spectrum antimicrobials	Antibiotic prescription review (Improve uptake of restricted antimicrobial form)	Adapt existing restricted antimicrobial form	Use of practice aids	O	Yes				

Learner improvement project activities (n=83)

Sector	Topic category	Improvement topic	Intervention or change idea	Intervention category	COM-B	Secured input or support from colleagues to undertake QI project	Collected data that improved understanding of problem	Gained new learning from implementing and evaluating a test of change (PDSA cycle)	Shared information and awareness about initiative outside of core team	Educated others to develop knowledge skills, attitudes
Secondary Care	48-72 hour review	72-hour review (Improve documentation of 72-hour review)	Reminder prompt at daily board round	Prompts and reminders	M					
Secondary Care	Antibiotic wastage	Antibiotic wastage in PD peritonitis patients	Implement/change procedure(s)	Guideline or procedure	O	Yes				
Secondary Care	Antibiotic duration	Duration of antibiotic therapy on vascular ward	Regular feedback during ward rounds	Prospective audit and feedback	Multiple	Yes				
Secondary Care	Antibiotic duration	Documentation of antibiotic duration on EPMA	Electronic rules/restrictions	Restriction	O	Yes				
Secondary Care	Surgical prophylaxis	Surgical prophylaxis (Reduce inappropriate prolonged duration prescribing)	Academic detailing/education	Education and promotion	C					

Learner improvement project activities (n=83)

Sector	Topic category	Improvement topic	Intervention or change idea	Intervention category	COM-B	Secured input or support from colleagues to undertake QI project	Collected data that improved understanding of problem	Gained new learning from implementing and evaluating a test of change (PDSA cycle)	Shared information and awareness about initiative outside of core team	Educated others to develop knowledge skills, attitudes
Secondary Care	Antibiotic allergy	Antibiotic allergy (Improve documentation of penicillin allergy in EPMA)	EPMA nudge prompts	Prompts and reminders	M	Yes				
Secondary Care	Lower respiratory tract infections	CAP (Improve guideline compliance)	Review local guidance	Guideline or procedure	O					
Secondary Care	Other	Antibiotic utilisation monitoring				Yes				
Secondary Care	48-72 hour review	72-hour review in cardiac surgery paediatric patients	Regular joint prescription review between pharmacist and prescribers	Prospective audit and feedback	Multiple		Yes			
Secondary Care	48-72 hour review	72-hour review	Prospective audit and feedback facilitated by mobile app	Prospective audit and feedback	Multiple	Yes				

Learner improvement project activities (n=83)

Sector	Topic category	Improvement topic	Intervention or change idea	Intervention category	COM-B	Secured input or support from colleagues to undertake QI project	Collected data that improved understanding of problem	Gained new learning from implementing and evaluating a test of change (PDSA cycle)	Shared information and awareness about initiative outside of core team	Educated others to develop knowledge skills, attitudes
Secondary Care	Documentation of indication	Documenting indication								
Secondary Care	Other	Compliance with sepsis 6 bundle	Education	Education and promotion	C	Yes	Yes			
Community pharmacy	Patient understanding of antimicrobials	Patient adherence with antibiotic prescription instructions	Revising standard operating procedures- antibiotic labelling and patient counselling	Guideline or procedure	C	Yes	Yes	Yes	Yes	Yes
Community pharmacy	UTI	Quality of antibiotic counselling and prescription review for UTI	Implement Community Pharmacy Checklist	Use of practice aids	C	Yes	Yes		Yes	Yes

Learner improvement project activities (n=83)

Sector	Topic category	Improvement topic	Intervention or change idea	Intervention category	COM-B	Secured input or support from colleagues to undertake QI project	Collected data that improved understanding of problem	Gained new learning from implementing and evaluating a test of change (PDSA cycle)	Shared information and awareness about initiative outside of core team	Educated others to develop knowledge skills, attitudes
Primary Care	Broad spectrum antimicrobials	Co-amoxiclav prescribing	Regular prospective audit and feedback; dissemination of updated formulary	Prospective audit and feedback	Multiple	Yes	Yes		Yes	Yes
Primary Care	Broad spectrum antimicrobials	Co-amoxiclav prescribing	Electronic clinical decision support (TARGET, consultation template); audit and feedback; introduce incentive scheme	Clinical decision support	C					
Primary Care	UTI	Nitrofurantoin UTI prophylaxis	TBC			Yes	Yes			

Learner improvement project activities (n=83)

Sector	Topic category	Improvement topic	Intervention or change idea	Intervention category	COM-B	Secured input or support from colleagues to undertake QI project	Collected data that improved understanding of problem	Gained new learning from implementing and evaluating a test of change (PDSA cycle)	Shared information and awareness about initiative outside of core team	Educated others to develop knowledge skills, attitudes
Secondary Care	Antibiotic dosing	Quality of IV antibiotic dosing in paediatrics	TBC				Yes		Yes	
Secondary Care	Other	Outpatient microbiological during outpatient surgical follow	Checklist and standard operating procedure	Guideline or procedure	C	Yes	Yes		Yes	Yes
Secondary Care	Lower respiratory tract infections	Antibiotic prescribing in CAP	Education and posters	Education and promotion	C	Yes		Yes	Yes	Yes
Secondary Care	Surgical prophylaxis	Surgical prophylaxis	Change local guidelines; education	Guideline or procedure	O	Yes	Yes	Yes	Yes	Yes

Learner improvement project activities (n=83)

Sector	Topic category	Improvement topic	Intervention or change idea	Intervention category	COM-B	Secured input or support from colleagues to undertake QI project	Collected data that improved understanding of problem	Gained new learning from implementing and evaluating a test of change (PDSA cycle)	Shared information and awareness about initiative outside of core team	Educated others to develop knowledge skills, attitudes
Secondary Care	Antibiotic allergy	Documentation of antibiotic indication and allergy status	TBC							
Secondary Care	Other	Ciprofloxacin prescribing for SBP prophylaxis	New procedure to flag review of patients on prophylaxis for more than 2 years	Guideline or procedure	M	Yes	Yes		Yes	
Secondary Care	Antibiotic dosing	Quality of teicoplanin dosing	TBC				Yes			
Secondary Care	Diagnostic stewardship	Use of urine dipsticks for UTI diagnosis	Education and restriction of dipstick use by nursing staff	Restriction	Multiple	Yes	Yes	Yes	Yes	Yes

Learner improvement project activities (n=83)

Sector	Topic category	Improvement topic	Intervention or change idea	Intervention category	COM-B	Secured input or support from colleagues to undertake QI project	Collected data that improved understanding of problem	Gained new learning from implementing and evaluating a test of change (PDSA cycle)	Shared information and awareness about initiative outside of core team	Educated others to develop knowledge skills, attitudes
Secondary Care	Antibiotic dosing	Antibiotic dosing on surgical ward	Introduce dose banding chart	Use of practice aids	C	Yes	Yes	Yes	Yes	Yes
Secondary Care	UTI	Quality of UTI antibiotic prescribing	Education, UTI prompt cards and prescription form changes	Use of practice aids	Multiple					
Secondary Care	Other	Central line management in OPAT service	Communicatio n checklist	Use of practice aids	C	Yes	Yes		Yes	Yes
Secondary Care	Surgical prophylaxis	Surgical prophylaxis in elective orthopaedic procedures	Education and posters	Education and promotion	C	Yes	Yes			

Learner improvement project activities (n=83)

Sector	Topic category	Improvement topic	Intervention or change idea	Intervention category	COM-B	Secured input or support from colleagues to undertake QI project	Collected data that improved understanding of problem	Gained new learning from implementing and evaluating a test of change (PDSA cycle)	Shared information and awareness about initiative outside of core team	Educated others to develop knowledge skills, attitudes
Secondary Care	Surgical prophylaxis	Prolonged antibiotic surgical prophylaxis in elective surgeries	Electronic prescribing template	Restriction	O	Yes	Yes		Yes	
Secondary Care	Documentation of indication	Documentation of indication and guideline compliance	TBC				Yes			
