# Supplementary material for: Outcomes of a National, Cross-Sector Antimicrobial Stewardship Training Initiative for Pharmacists in England
Source: Pharmacy (Basel). 2021 Oct 10;9(4):165. doi: 10.3390/pharmacy9040165 (PMC8544733; doi:10.3390/pharmacy9040165)
Supplement: Supplementary file 1 [file pharmacy-09-00165-s001.zip › Supplementary materials (S6)- learner feedback survey responses (learner experience and value of training).pdf]

**Summary of survey responses**		
	Learner experience	
Cohort 1 (n=10):	Cohort 2 (n=39)	Cohort 3 (n=13)
Rate your satisfaction with the training on a scale of 1 to 10<br>(1 = "very dissatisfied" and 10 = "very satisfied")<br><br>Average satisfaction rating: 8.3 out of 10	Satisfaction with the training:<br><br>• "Very satisfied" (26%)<br>• "Satisfied" (59%)<br>• "Neither satisfied or dissatisfied" (10%)<br>• "Dissatisfied" (2.5%)<br>• "Very dissatisfied" (2.5%)	Overall experience of the training:<br><br>• "Very good" (92%)<br>• "Good" (8%)<br>• "Average" (0%)<br>• "Poor" (0%)<br>• "Very poor" (0%)
	Value of the training	
Cohort 1 (n=10):	Cohort 2 (n=39)	Cohort 3 (n=13)
Would you recommend the training to a colleague?<br><br>• Yes (80%)<br>• Maybe (20%)<br>• No (0%)	Would you recommend the training to a colleague?<br><br>• Yes (90%)<br>• Maybe (10%)<br>• No (0%)	"Learning outcomes were relevant and appropriate to my practice"<br><br>• Strongly agree (62%)<br>• Agree (38%)<br>• Neither agree or disagree (0%)<br>• Disagree (0%)<br>• Strongly disagree (0%)<br><br>"Participating in this training was a valuable use of my time"<br><br>• Strongly agree (85%)<br>• Agree (15%)<br>• Neither agree or disagree (0%)<br>• Disagree (0%)<br>• Strongly disagree (0%)
